# Supplementary material for: BRAF activation by metabolic stress promotes glycolysis sensitizing NRASQ61-mutated melanomas to targeted therapy
Source: Nat Commun. 2022 Nov 19;13:7113. doi: 10.1038/s41467-022-34907-0 (PMC9675737; doi:10.1038/s41467-022-34907-0)
Supplement: Supplementary file 1 — Supplementary information [file 41467_2022_34907_MOESM1_ESM.pdf]

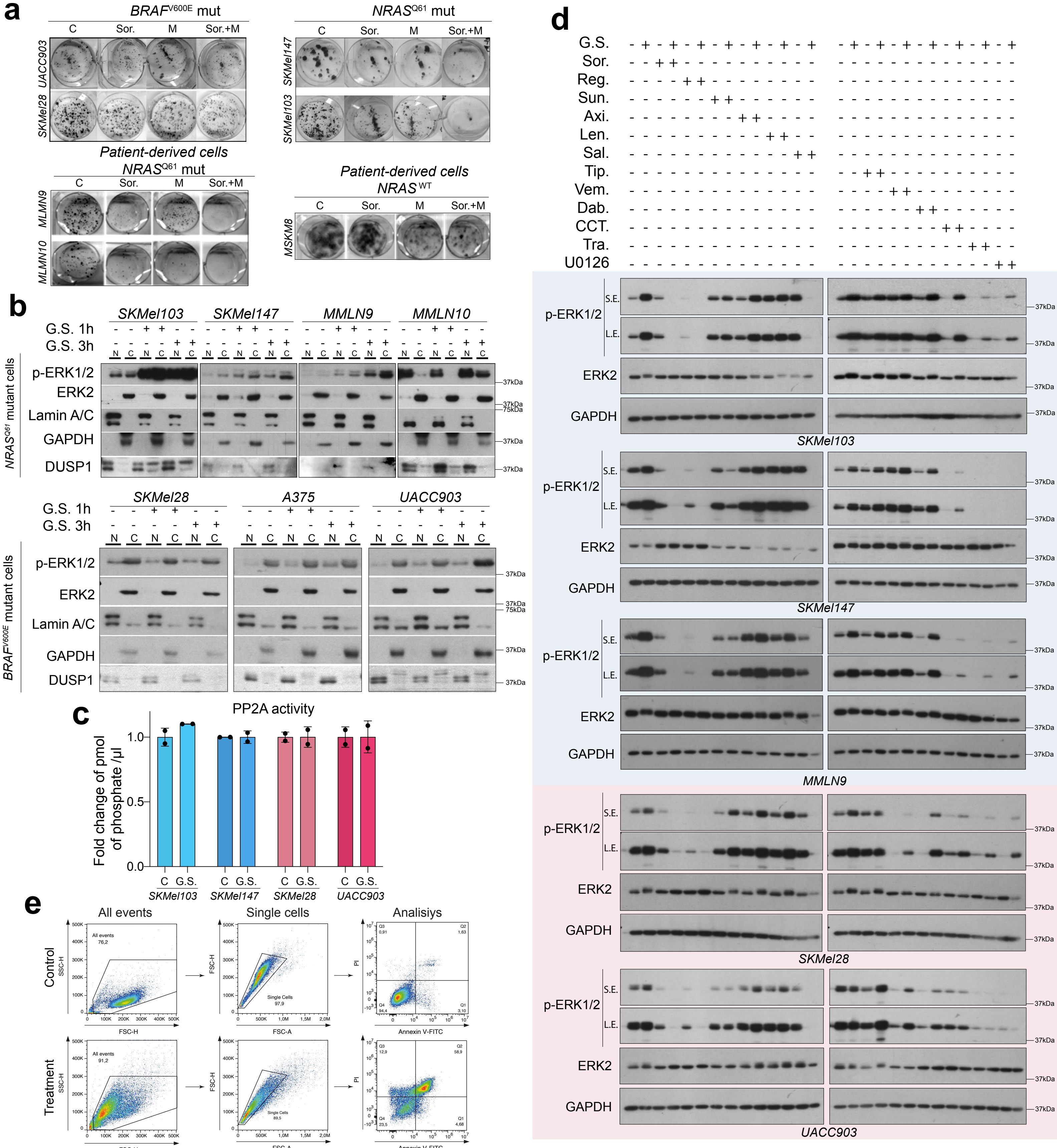

Suplementary Figure 1: Sensitization of *NRAS* mutant melanomas to sorafenib upon metabolic stress and subcellular localization of p-ERK1/2 upon metabolic stress. (**a**) Colony formation assay of *NRAS*^{Q61} mutant melanoma cells including patient-derived cells (MSKM8,MMLN9 and MMLN10) and *BRAF*^{V600E} mutant cells exposed to metformin (M) and sorafenib (Sor.). (**b**) Representative western-blot showing p-ERK1/2 amounts in the nucleus or cytoplasm in response to G.S. in *NRAS*^{Q61}mutant melanoma cells including patient-derived cells (MMLN9, MMLN10) and *BRAF*^{V600E} mutant cells. Lamin A/C and GAPDH are used as nuclear and cytoplasmic markers.(**c**) Graph showing PP2A activity 4h after G.S. Mean ± SD (**d**) Representative western-blot showing the effect of the indicated inhibitors on ERK1/2 activation. Glucose deprivation (G.S.). *NRAS*^{Q61} mutant cells are in blue background and *BRAF*^{V600E}mutant cell lines are in pink background. Sorafenib (Sor.) (inhibitor of BRAF^{V600E}, BRAF,CRAF, VEGFR2, PDGFRβ, FLT3, cKIT, and FGFR1); Regorafenib (Reg.)(inhibitor of VEGFR1,VEGFR2, VEGFR3, PDGFRβ, Kit (c-Kit), RET (c-RET) and CRAF, BRAF^{V600E} and BRAF); Sunitinib (Sun.) (inhibitor of FLT3, c-KIT, PDGFRβ, VEGFR2, LCK, FGFR1, EGFR, CDK2, MET, IGFR1, ABL and SRC); Axitinib (Axi.) (inhibitor of VEGFR1, VEGFR2, VEGFR3, PDGFRβ and c-Kit); Lenvatinib (Len.) (inhibitor of VEGFR1, VEGFR2, VEGFR3, PDGFRα, FGFR1-4, RET (cRET) and c-Kit); Salirasib (Sal.) (inhibits RAS methylation); Tipifarnib (Tip.)(farnesyl-transferase (FTase) inhibitor, mostly HRAS); Vemurafenib (Vem.) (inhibitor of BRAF^{V600E}, BRAF), Dabrafenib (Dab.) (inhibitor of BRAF^{V600E}, BRAF); CCT196969 (CCT.) (inhibitor of BRAF^{V600E}, BRAF, CRAF, Src andLck); Trametinib (Tra.) (inhibitor of MEK1/2) and U0126 (inhibitor of MEK1/2). (**e**) Gating strategy for apoptosis analysis. Number of biologicaly independent experiments: *n*=3 in(**b**) and *n*=2 in (**c**) and (**d**). C= control; G.S.= Glucose starvation.

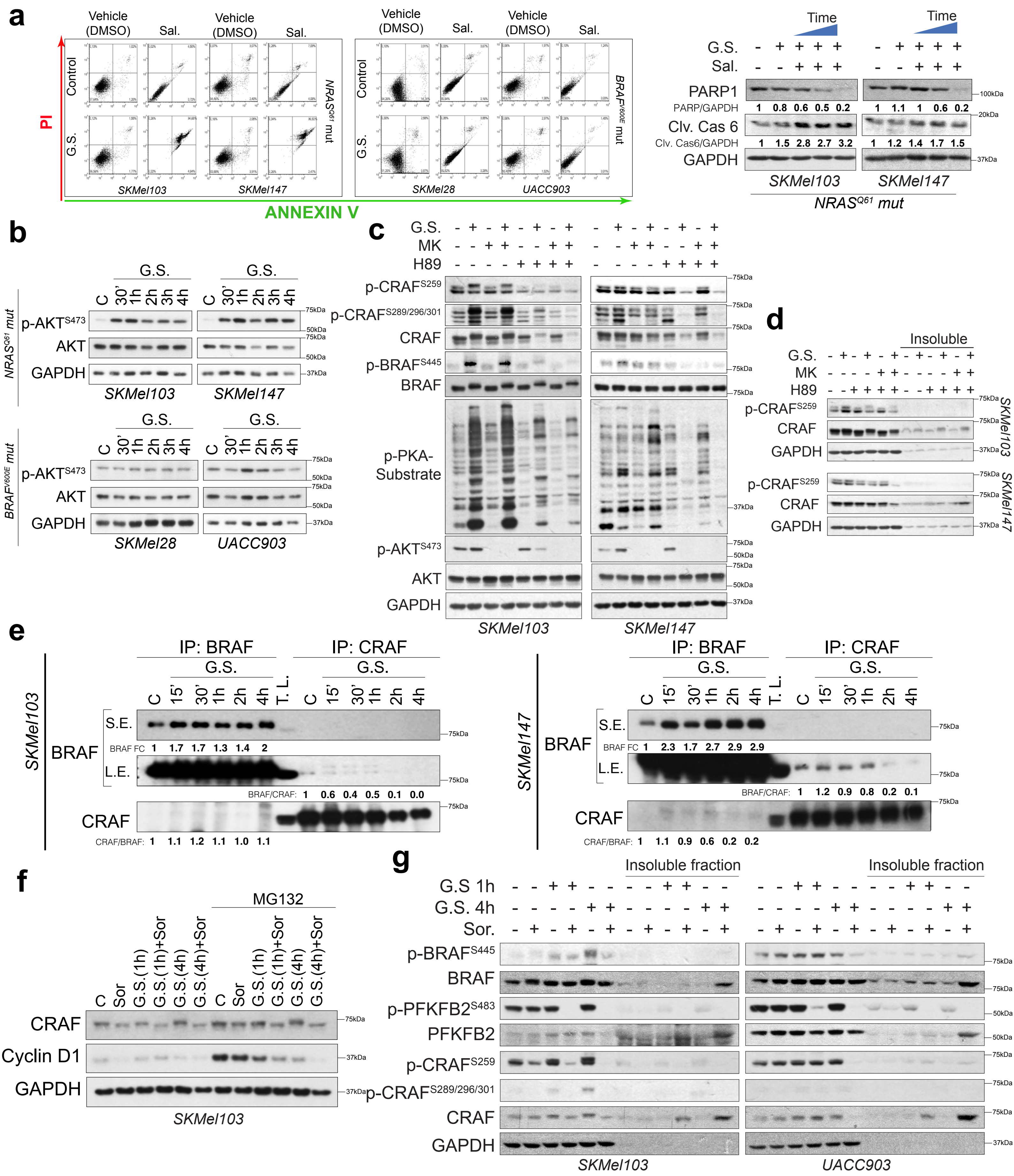

Supplementary Figure 2: RAS inhibition upon glucose starvation (G.S.) lead to cell death and G.S. induces the AKT and PKA-dependent switch of RAF isoform use. (**a**) Representative experiment of apoptosis detection by flow cytometry analysis of *$BRAF^{V600E}$* and *$NRAS^{Q61}$* mutant melanoma cells stained with propidium iodide (PI) and Annexin-V-EGFP. Cells were treated for 4h with salirasib (Sal.) in the presence or absence of glucose (G.S.). On the right, Immunoblot showing the indicated apoptotic markers upon the same conditions. (**b**) Representative western-blot showing AKT activation under G.S. in *$NRAS^{Q61}$* and *$BRAF^{V600E}$* mutant melanoma cell lines. (**c**) Representative western-blot showing the effects of PKA and AKT activation upon G.S. on CRAF and BRAF phosphorylation in the presence of MK=MK2206, H-89 inhibitors. (**d**) Ilustrative immunoblot showing CRAF translocation to the NP-40-insoluble fraction after indicated treatments. (**e**) Representatitve immunoblot showing the immunoprecipitated RAF proteins isoforms from *SKMel103* and *SKMel147* cells in a time course manner upon G.S. S.E. short exposure, L.E. long exposure. Numbers indicate fold changes respect the control of either BRAF or BRAF/CRAF and CRAF/BRAF ratio. (**f**) Ilustrative western-blot showing the expression of CRAF in the presence and absence of the proteosome inhibitor MG132 upon G.S. and in combination with Sor. at the time points indicated. Cyclin D1 is showed as a control of MG132 activity and GAPDH as a loading control. (**g**) Representative experiment where total cell lysates of NP-40-soluble and -insoluble fractions were analyzed by western blotting against the indicated antibodies. Cells were subjected to G.S. for the indicated times in the presence or absence of Sor. A representative experiment is showed. Numbers show fold induction of the indicated proteins or ratios. Number of biogically independent experiments in (**a**), (**b**), (**c**), (**d**), (**e**), (**f**), and (**g**): *n*=3. C= control; G.S.= Glucose starvation; Sor.= Sorafenib.

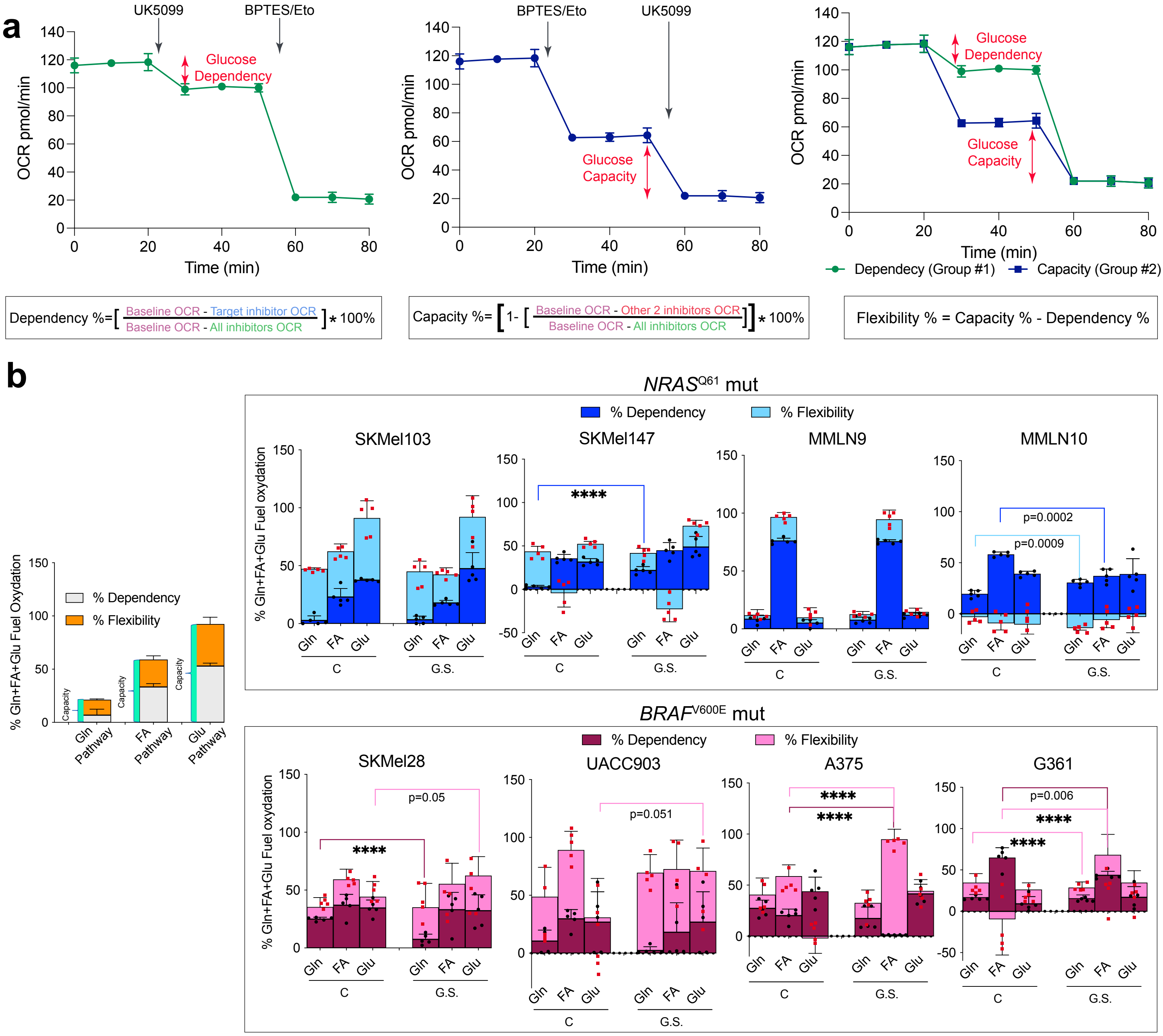

Supplementary Figure 3. In the absence of glucose *NRAS*^{Q61} mutant cells are less flexible than *BRAF*^{V600E} mutant cells using other energy fuel sources. (a) Explanatory graphs and equations for the calculation of fuel dependency, fuel capacity and fuel source flexibility. (**b**) Graphs representing percentage of dependency, capacity and flexibility of *NRAS*^{Q61} and *BRAF*^{V600E} mutant melanoma cells to oxydize glucose (Glu), long chain-fatty acids (FA) or glutamine (Gln) as mitochondrial source of fuel (explanatory graph on the left) under normal conditions (C) and under glucose deprivation (G.S.). Mean ± SD, n = 5 biologicaly independent experiments are represented , **** $p < 0.0001$; unpaired two-sided t test. Dark blue and deep red colors statistical lines compare dependencies, light blue and pink statistical lines compare flexibility.

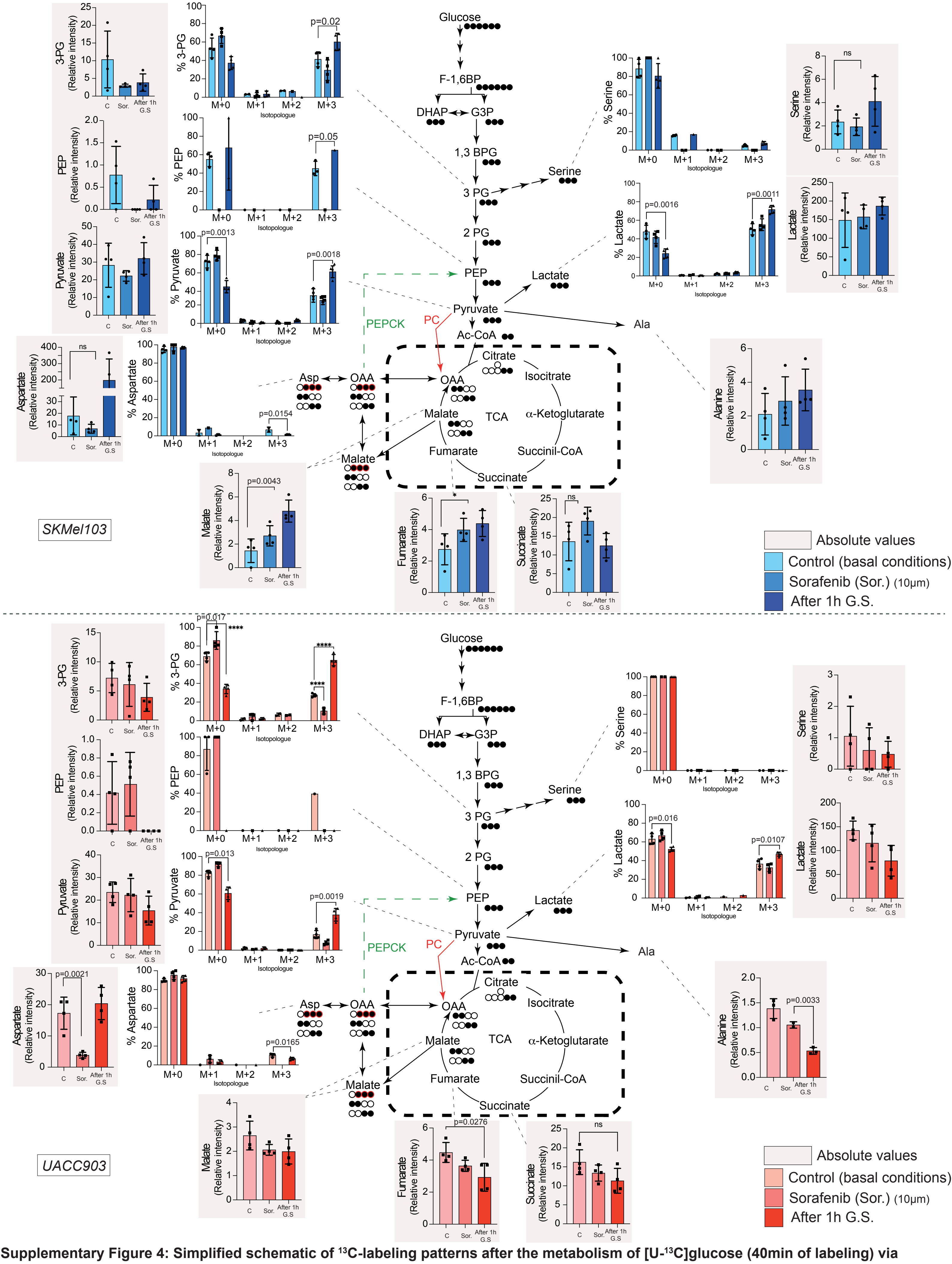

Supplementary Figure 4: Simplified schematic of ^{13}C-labeling patterns after the metabolism of [U-^{13}C]glucose (40min of labeling) via glycolysis and the TCA cycle. Empty circles, ^{12}C; black filled circle, ^{13}C; black filled circles with red ring, ^{13}C derived from pyruvate dexcarboxylase (PC). Graphs in white background represent the identified isotopologues derivates of the indicated intermediates. Graphs in grey background show absolute amount of derivatives (sum of all isotopologues) at the indicated conditions: C (clear blue and clear red)=Basal condition, Sor. (mild blue and mild red) Sorafenib in complete medium and after 1h glucose starvation (G.S). (dark blue and dark red)= labeling after 1h of glucose starvation. Experiments were performed in cuadruplicates. Mean ± SD are represented; **** p<0.001, (p-value, unpaired two-sided t-test).

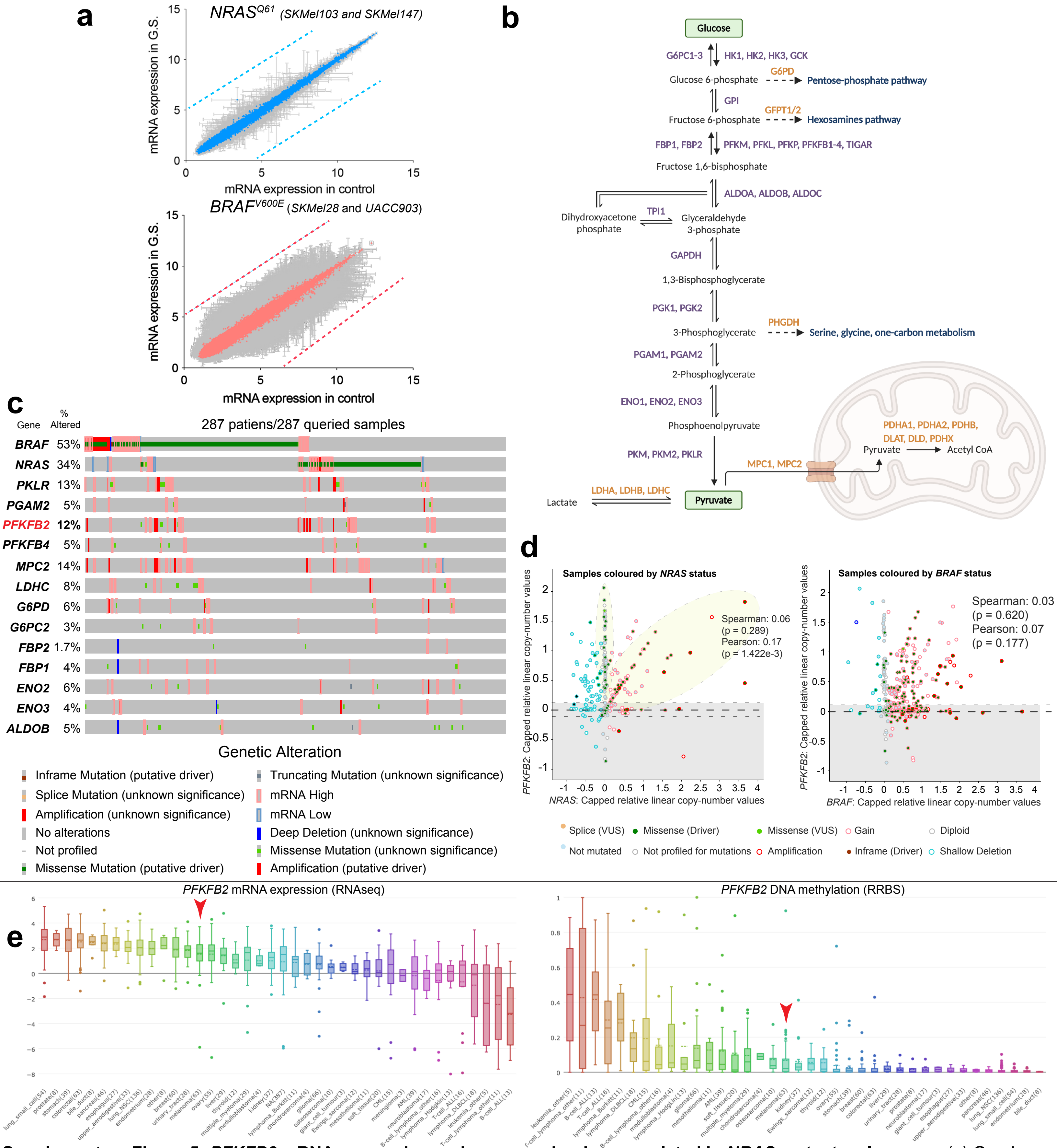

Supplementary Figure 5: *PFKFB2* mRNA expression and copy number is upregulated in *NRAS* mutant melanomas. (**a**) Graphs showing the correlation of the gene expressed values regulated in response to G.S. (G.S.vs control) in *NRAS*^{Q61} (*SKMel103* and *SKMel147*) and *BRAF*^{V600E} (SKMel28 and *UACC903*) mutant melanoma cells lines. *n*=3 biologicaly independent experiments per cell line and condition. Mean ± SD are represented. (**b**) Scheme of glycolysis showing the enzymes of interest interrrogated in figure 5b. (**c**) Genetic alterations in *NRAS* mutated cells-regulated genes in response to G.S. (Figure 5b) inTCGA melanoma dataset (TCGA Firehose legacy). (**d**) Graphs showing the correlation of copy number values of *PFKFB2* with *NRAS* and *BRAF* and the mutational status of samples in respect to *NRAS* and *BRAF*. p-values were calculated performing both Spearman and Pearson correlation. (**e**) *PFKFB2* mRNA expression and promoter methylation status in 63 melanoma cell lines and across to 40 different tumor types. Box-and-whisker plots show the distribution of mRNA expression for each subtype, ordered by the median *PFKFB2* expression or promoter methylation level (line), the interquatile range (box) and up to 1.5x the inter-quartile range (bars). Number of cell lines of each tumor type is shown within the parenthesis. Arrow shows the melanoma location. G.S.= Glucose starvation.

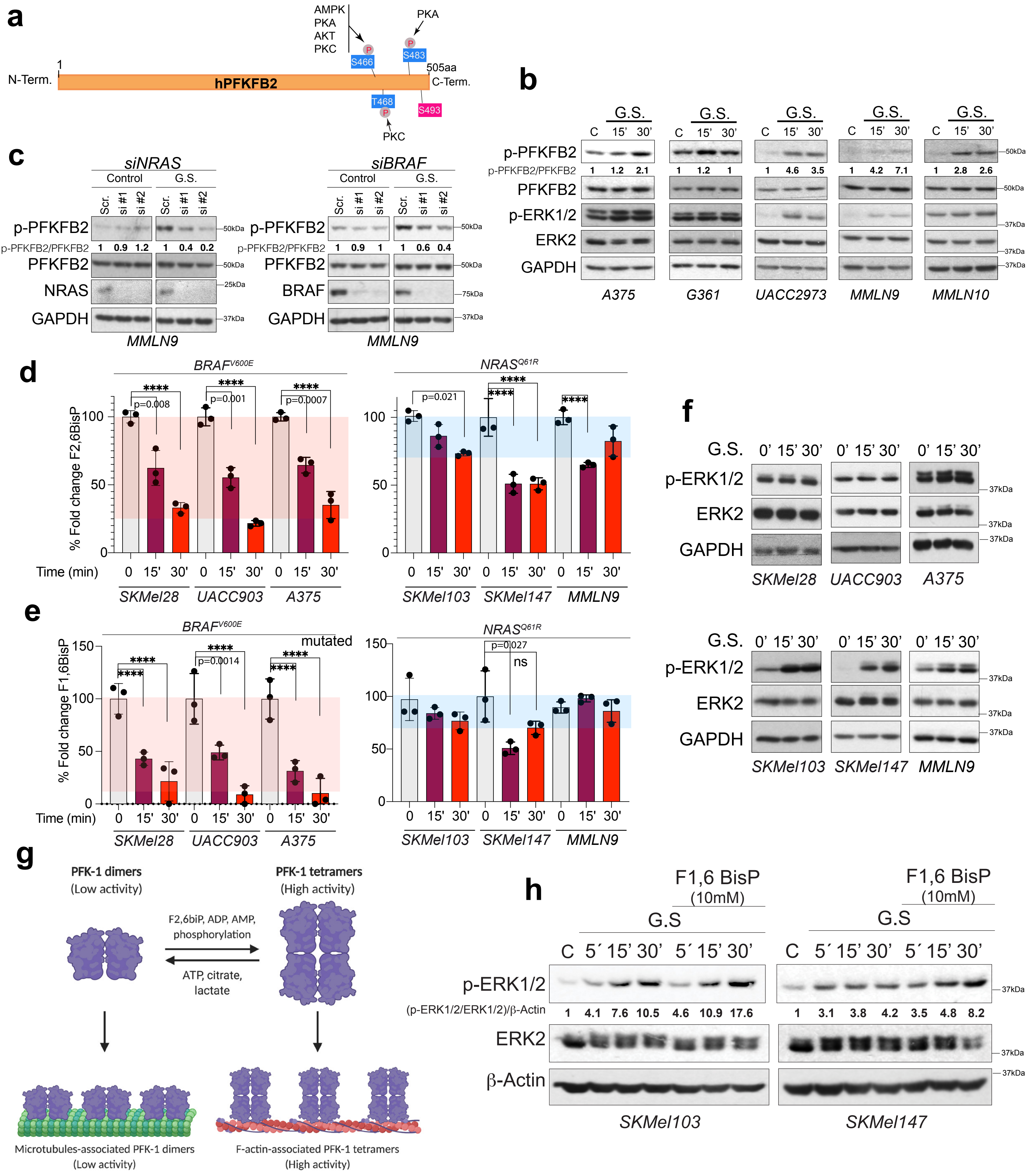

Supplementary Figure 6: **Glucose starvation (G.S.) induces PFKFB2^{S483} phosphorylation and metabolite changes at early time points.** (**a**) Human PFKFB2 phosphorylation residues. The kinases described to phosphorylate the depicted residues are shown (blue). (**b**) Representative western blot showing PFKFB2^{S483} phosphorylation after G.S. in the indicated melanoma cell lines. Fold induction respect the control is showed by numbers. (**c**) Representative immunoblots showing PFKFB2 phosphorylation regulation (S483) in response to metabolic stress G.S. in *NRAS* or *BRAF* knockdown *MMLN9* patient-derived cells. Graphs showing the variation in F2, 6BisP(**d**) and F1, 6BisP (**e**) upon G.S. at the indicated time points in three *BRAF*^{V600E} and three *NRAS*^{Q61} mutated melanoma cell lines including a patien-derived cell line (MMLN9). **** p<0.0001, (p-value, Mean ± SD; unpaired two-sided t-test). Painted red and blue areas represent the avereage fold change decreased among the cell lines (red *BRAF*^{V600E}-mutated and blue *NRAS*^{Q61}-mutated melanoma cells). (**f**) Representative western-blots showing the RAS pathway hyperactivation at the same time points in the assayed cell lines at (**d**) and (**e**). (**g**) Scheme showing the activation of PFK1 according to its homodimerization or homotetradimerization state and its association with cytoskeleton fibers. (**h**) Representative western-blots showing the activation of ERK1/2 in G.S. upon the addition of 10mM F1,6BisP at the indicated time points. Numbers show fold induction of the indicated ratios. *n*=3 biologicaly independent experiments in (**b**), (**c**), (**d**), (**e**), (**f**) and (**h**). G.S.=Glucose starvation.

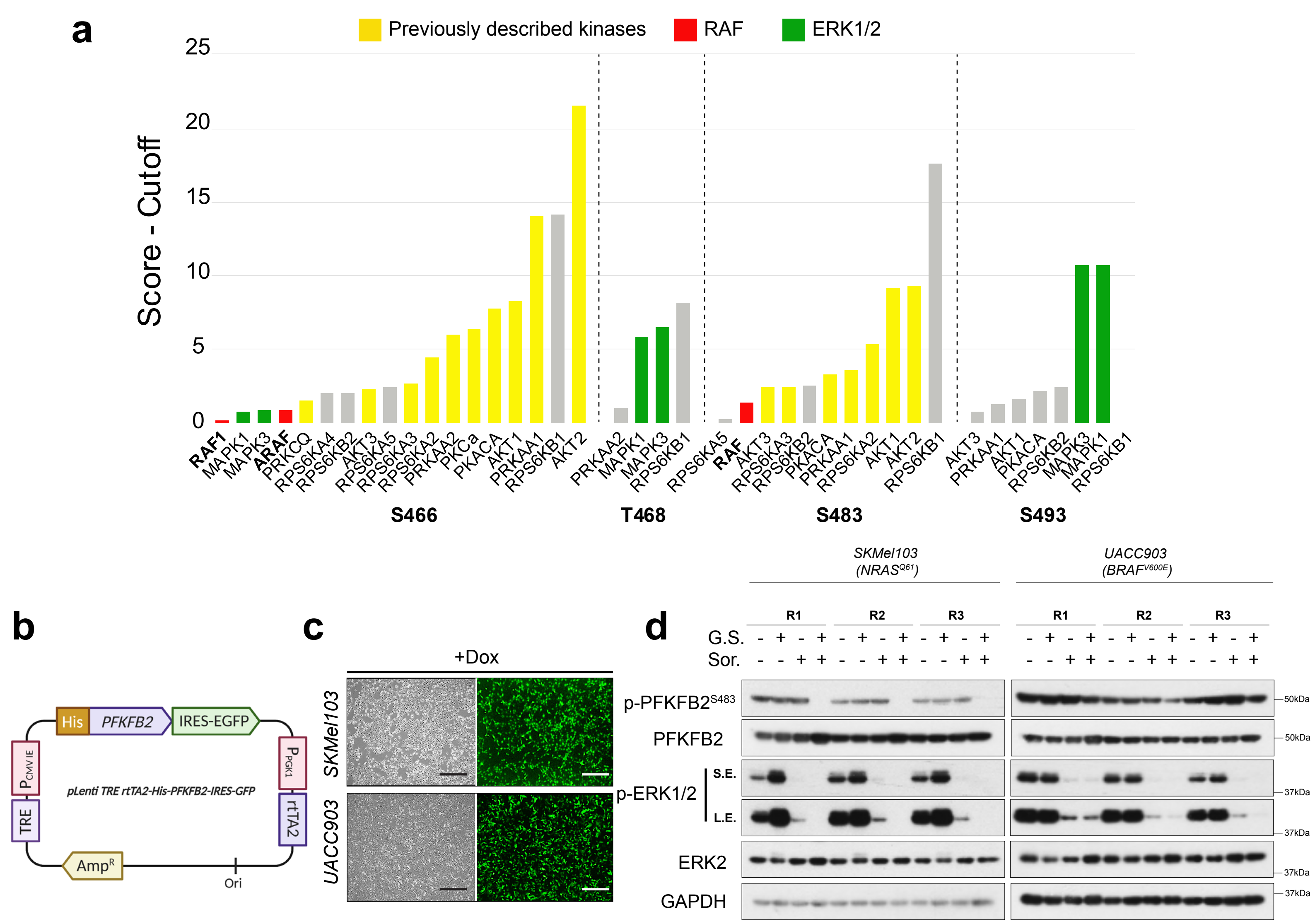

e

Protein	SKMel103 control					SKMel103 G.S.				SKMel103 Sor.				SKMel103 G.S.+ Sor			
	MW [kDa]	Score	#Peptides	SC [%]	# Spec Counts	Score	#Peptides	SC [%]	# Spec Counts	Score	#Peptides	SC [%]	# Spec Counts	Score	#Peptides	SC [%]	# Spec Counts
PFK/FBPase 2	58.4	933.8	32	61.2	47	2719.6	60	79.2	135	796.7	31	61.6	43	1782	47	74.3	93
HSP 90-alpha	84.6	311.5	13	21.6	13	368.7	17	24.7	19	455.2	20	28.3	22	486.8	20	26.2	21
HSP 90-beta	83.2	314.9	14	24	14	407.3	15	23.9	17	409.9	16	25.8	19	443.1	16	25.1	18
CDC37	44.4	183.3	10	29.4	10	136.4	6	20.9	7	177.4	9	26.7	10	230.3	10	29.4	10
AMPK- alpha-1 4	64	n.d	-	-	-	217.3	9	24	11	51.1	2	3.6	2	98.3	6	12.3	6
AMPK- alpha-2	62.3	n.d	-	-	-	142.1	3	8	3	n.d.	-	-	-	n.d	-	-	-
14-3-3 beta/alpha	28.1	51.4	2	8.9	3	91.4	4	17.1	5	112.2	4	17.1	5	37.6	3	12.6	3
14-3-3 theta	27.7	100.3	3	16.7	3	148.8	6	25.3	7	107	5	20.8	5	81.6	4	20.8	4
14-3-3 gamma	28.3	n.d.	-	-	-	58.9	2	8.9	2	n.d.	-	-	-	36.5	2	8.9	2
ERK2	41.4	n.d.	-	-	-	250.6	12	45	17	n.d.	-	-	-	127.3	9	31.1	11
PFK/FBPase 3	59.6	n.d.	-	-	-	304	8	18.3	13	n.d.	-	-	-	147.5	7	13.8	10
β–Actin	41.7	n.d.	-	-	-	483.5	13	49.3	21	69.8	3	8.8	4	206.5	8	27.2	11

		UACC903 Control				UACC903 G.S.				UACC903 Sor.				UACC903 G.S.+ Sor.			
	MW [kDa]	Score	#Peptides	SC [%]	# Spec Counts	Score	#Peptides	SC [%]	# Spec Counts	Score	#Peptides	SC [%]	# Spec Counts	Score	#Peptides	SC [%]	# Spec Counts
PFK/FBPase 2	58.4	1099	37	66.3	53	1229.4	38	67.5	58	883.5	34	64.2	51	899.1	33	59	47
HSP 90-alpha	84.6	168.1	6	11.2	6	291.4	11	18.2	11	281.7	11	16.3	12	298.9	11	16.4	12
HSP 90-beta	83.2	306.7	13	23.5	13	388	17	29	17	422.5	20	31.5	21	463.3	17	26	19
CDC37	44.4	105.1	6	18.5	6	135.9	7	24.1	7	165.6	8	26.7	9	186.4	7	24.1	8
AMPK- alpha-1 4	64	n.d	-	-	-	n.d	-	-	-	n.d	-	-	-	29.8	1	1.8	1
AMPK- alpha-2	62.3	n.d	-	-	-	n.d	-	-	-	n.d	-	-	-	n.d.	-	-	-
14-3-3 beta/alpha	n.d.	n.d.	-	-	-	59.7	2	8.9	2	n.d.	-	-	-	n.d.	-	-	-
14-3-3 theta	97.3	2	9	2	-	85.2	2	9	2	73.8	2	9	2	141	6	25.3	6
14-3-3 gamma	28.3	n.d.	-	-	-	n.d.	-	-	-	n.d.	-	-	-	n.d.	-	-	-
ERK2	41.4	-	-	-	-	-	-	-	-	-	-	-	-	30.5	2	4.7	2
PFK/FBPase 3	59.6	-	-	-	-	-	-	-	-	-	-	-	-	n.d.	-	-	-
β–Actin	41.7	53.2	3	8.8	3	136.6	4	13.1	4	71.7	3	8.8	3	127.5	4	13.1	5

Supplementary Figure 7: Human PFKFB2 phosphorylation residues. (**a**) Graph showing the putative residues to be phosphorylated by the top (high stringency, Score-Cutoff>2) kinases according to the residue sequence context after human PFKFB2 sequence analysis using GPS 5.0. (**b**) Map of the lentiviral construct containing the His-tagged-*PFKFB2* isoform. (**c**) Representative pictures of induced infected cells. Bars represent 400μm (**d**) Western blot showing the response to G.S. and Sor. of *NRAS*^{Q61} and *BRAF*^{V600E} mutant induced infected cells. These triplicates were the samples used for the mass spectometry analysis. S.E short exposure; L.E. long exposure. (**e**) Table showing the proteins identified by mass spectometry that have been described in the literature to interact with PFKFB2 (Ocre background) and the new ones likely involved in the studied mechanism (garnet background). G.S.= Glucose starvation; Sor.= Sorafenib.

a

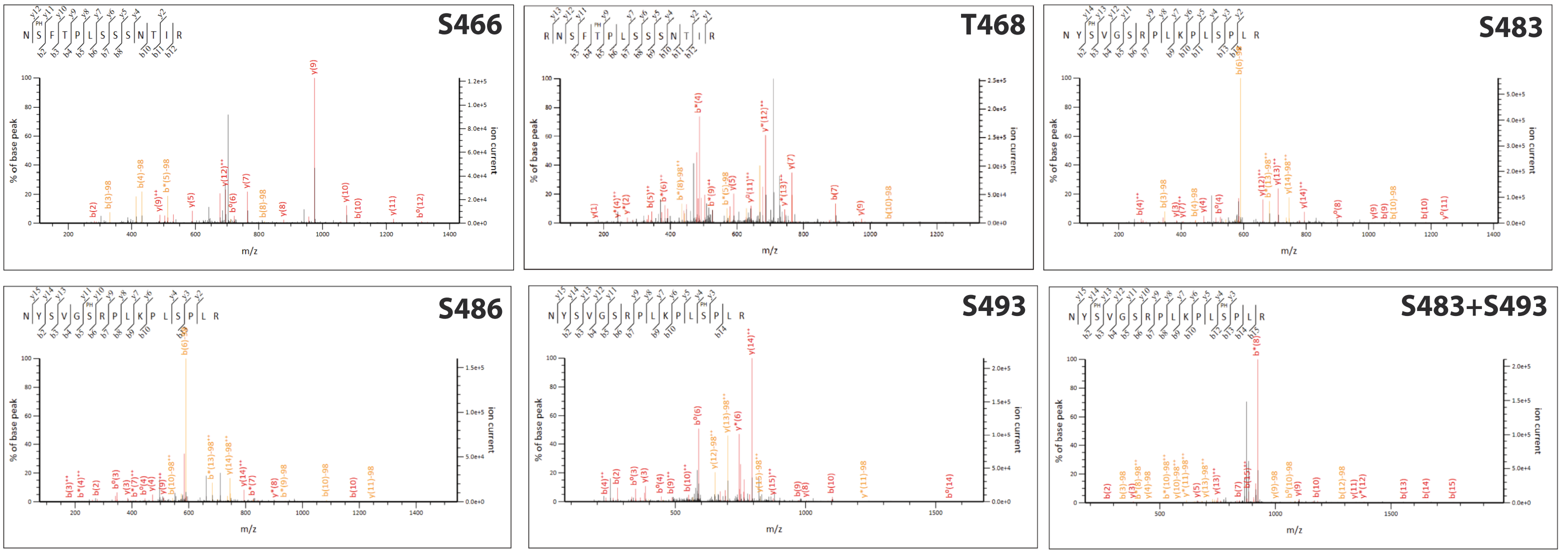

b

Residue	Score	Mr (calc.)	Delta	Sequence	Site Analysis
**S466**	53,7	1502,6766	-0,0001	NSFTPLSSSNTIR	**Phospho S2, 99.12%**
	33,1	1502,6766	-0,0001	NSFTPLSSSNTIR	Phospho T4 0.86%
	16,1	1502,6766	-0,0001	NSFTPLSSSNTIR	Phospho S7 0.02%
**S466+T468**	54.6	1.658,7777	-0.0000001	RNSFTPLSSSNTIR	**Phospho S3, 85.85%**
	46.7	1.658,7777	-0.0000001	RNSFTPLSSSNTIR	**Phospho T5, 13.99%**
	25.3	1.658,7777	-0.0000001	RNSFTPLSSSNTIR	Phospho S8 0.10%
	20.3	1.658,7777	-0.0000001	RNSFTPLSSSNTIR	Phospho S9 0.03%
	15.0	1.658,7777	-0.0000001	RNSFTPLSSSNTIR	Phospho S10 0.01%
	12.2	1.658,7777	-0.0000001	RNSFTPLSSSNTIR	Phospho T12 0.00%
**S466+T468**	26.7	1.658,7777	-0.0013	RNSFTPLSSSNTIR	**Phospho S3, 49.05%**
	26.6	1.658,7777	-0.0013	RNSFTPLSSSNTIR	**Phospho T5, 49.85%**
**S483**	34,7	1862,9767	0.0011	NYSVGSRPLKPLSPLR	**Phospho S3, 95.97%**
	20,9	1862,9767	0.0011	NYSVGSRPLKPLSPLR	Phospho S6 3.99%
**S486**	20,4	1862,9767	0.0007	NYSVGSRPLKPLSPLR	Phospho S6 50.92%
**S493**	27,3	1862,9767	0.0029	NYSVGSRPLKPLSPLR	**Phospho S13, 99.53%**
	2,4	1862,9767	0.0029	NYSVGSRPLKPLSPLR	Phospho S6 0.32%
**S483+S493**	27.6	1.942,9431	0.017	NYSVGSRPLKPLSPLR	**Phospho S3, S13 88.19%**
	17.3	1.942,9431	0.017	NYSVGSRPLKPLSPLR	Phospho S3, S6 8,29%
	13.6	1.942,9431	0.017	NYSVGSRPLKPLSPLR	Phospho S6, S13 3,52%

c

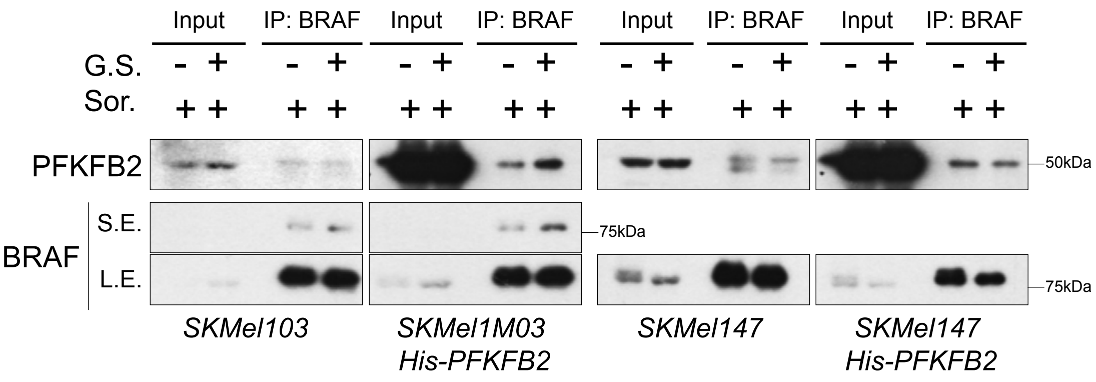

Supplementary Figure 8: Phopshopeptide analysis of PFKFB2 by mass spectrometry. (a) Phosphopeptide fragmentation spectrums of the identified phosphorylated residues. **(b)** Table showing the matches for the queries and percentages of the phosphorylated residue at the indicated site of the analysis. **(c)** Representative immunoblots showing the binding of PFKFB2 to BRAF in parental and His-tagged-PFKFB2 infected cells, upon G.S.and in the presence or absence of Sor. *n*=2 independent biological experiments in the two different cel lines. S.E.= short exposure; L.E.= long exposure;G.S.=4h Glucose starvation; Sor.= Sorafenib

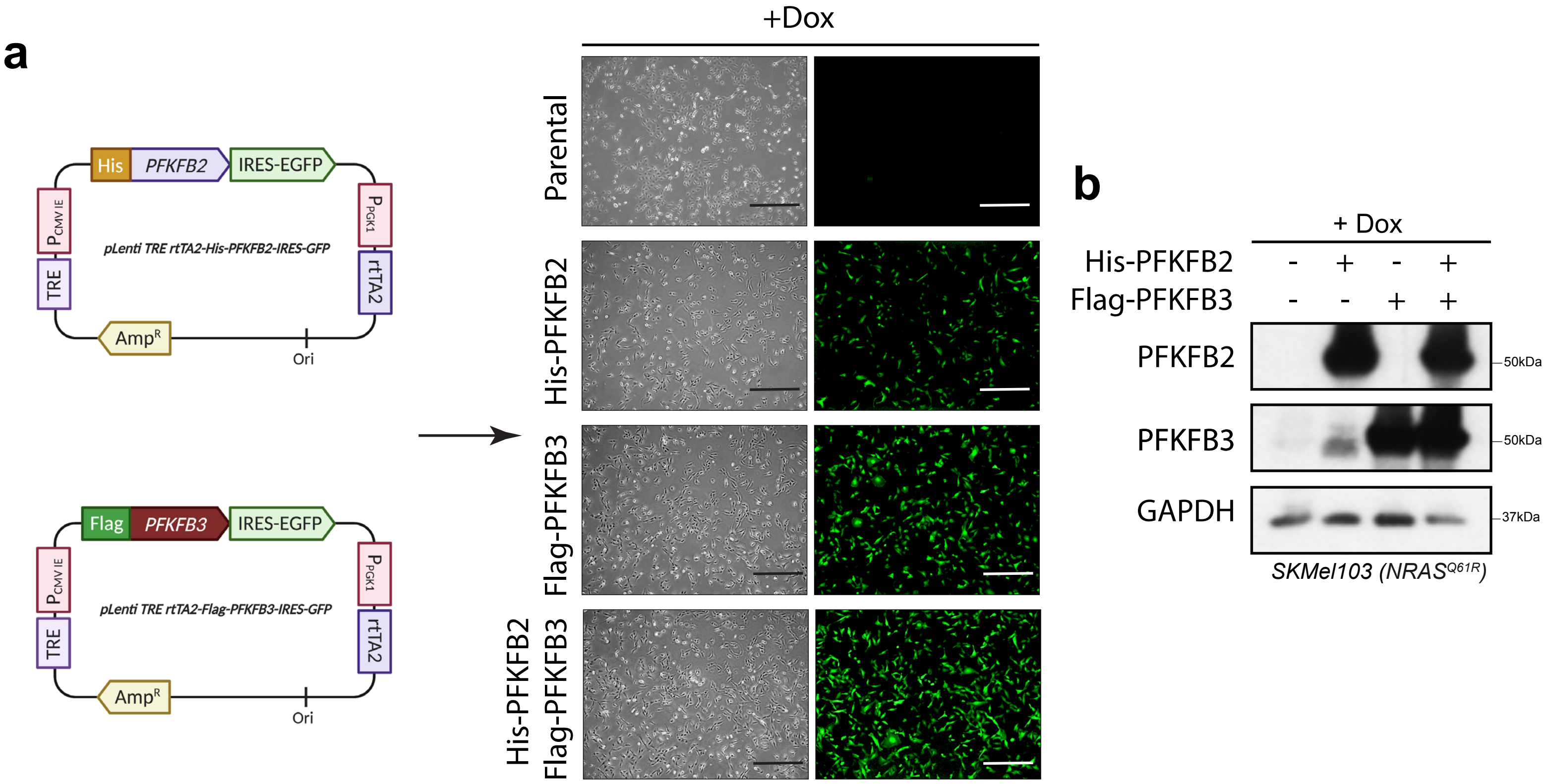

Supplementary Figure 9: PFKFB2 and PFKFB3 expressing cells. (**a**) Maps of the constructs expressing the tagged isoforms of PFKFB2 and PFKFB3. On the right, induced infected cells with the diffent constructs.Bars represent 400 µm. (**b**) Representative western-blot showing the expression of the indicated proteins. *n*=2 biologicaly independent experiments in (**a**) and (**b**).
