# Supplementary material for: BRAF activation by metabolic stress promotes glycolysis sensitizing NRASQ61-mutated melanomas to targeted therapy
Source: Nat Commun. 2022 Nov 19;13:7113. doi: 10.1038/s41467-022-34907-0 (PMC9675737; doi:10.1038/s41467-022-34907-0)
Supplement: Supplementary file 3 — Reporting Summary [file 41467_2022_34907_MOESM3_ESM.pdf]

nature portfolio

Corresponding author(s): Juan A. Recio

Last updated by author(s): Oct 31, 2022

Reporting Summary

Nature Portfolio wishes to improve the reproducibility of the work that we publish. This form provides structure for consistency and transparency in reporting. For further information on Nature Portfolio policies, see our Editorial Policies and the Editorial Policy Checklist.

Statistics

For all statistical analyses, confirm that the following items are present in the figure legend, table legend, main text, or Methods section.

n/a	Confirmed	
☐	☒	The exact sample size (*n*) for each experimental group/condition, given as a discrete number and unit of measurement
☐	☒	A statement on whether measurements were taken from distinct samples or whether the same sample was measured repeatedly
☐	☒	The statistical test(s) used AND whether they are one- or two-sided<br>*Only common tests should be described solely by name; describe more complex techniques in the Methods section.*
☒	☐	A description of all covariates tested
☒	☐	A description of any assumptions or corrections, such as tests of normality and adjustment for multiple comparisons
☐	☒	A full description of the statistical parameters including central tendency (e.g. means) or other basic estimates (e.g. regression coefficient) AND variation (e.g. standard deviation) or associated estimates of uncertainty (e.g. confidence intervals)
☐	☒	For null hypothesis testing, the test statistic (e.g. *F*, *t*, *r*) with confidence intervals, effect sizes, degrees of freedom and *P* value noted<br>*Give P values as exact values whenever suitable.*
☒	☐	For Bayesian analysis, information on the choice of priors and Markov chain Monte Carlo settings
☐	☒	For hierarchical and complex designs, identification of the appropriate level for tests and full reporting of outcomes
☒	☐	Estimates of effect sizes (e.g. Cohen's *d*, Pearson's *r*), indicating how they were calculated

Our web collection on statistics for biologists contains articles on many of the points above.

Software and code

Policy information about availability of computer code

Data collection	Seahorse technology, MASCOT, 3DHistech software, GeneChip™ Scanner 3000 Instrument, FACSCalibur (Becton Dickinson), RACE 1300 Series GC coupled to an Orbitrap Exactive mass spectrometer (Thermo Scientific)
Data analysis	ImageJ v1.53a, GraphPad, Prism 9.0, FlowJo v10, GPS 5.0 software, BioConductor package oligo, R studio (v.1.2.5001), Mascot, ProteinScape software, Metascape, Heatmapper

For manuscripts utilizing custom algorithms or software that are central to the research but not yet described in published literature, software must be made available to editors and reviewers. We strongly encourage code deposition in a community repository (e.g. GitHub). See the Nature Portfolio guidelines for submitting code & software for further information.

Data

Policy information about availability of data

All manuscripts must include a data availability statement. This statement should provide the following information, where applicable:

- Accession codes, unique identifiers, or web links for publicly available datasets
- A description of any restrictions on data availability
- For clinical datasets or third party data, please ensure that the statement adheres to our policy

The gene expression microarray data sets reported in this article were deposited in ArrayExpress with accession number E-MTAB-10921. The mass spectrometry proteomics data have been deposited to the ProteomeXchange Consortium via the PRIDE partner repository with the dataset identifier PXD028297. Gene alteration and mRNA expression/DNA methylation data used in this study are publicly available at cBioPortal (https://www.cbioportal.org) and CCLE databases(https://sites.broadinstitute.org/ccle), respectively.

Field-specific reporting

Please select the one below that is the best fit for your research. If you are not sure, read the appropriate sections before making your selection.

- [x] Life sciences
- [ ] Behavioural & social sciences
- [ ] Ecological, evolutionary & environmental sciences

For a reference copy of the document with all sections, see nature.com/documents/nr-reporting-summary-flat.pdf

Life sciences study design

All studies must disclose on these points even when the disclosure is negative.

Sample size	The sample sizes were determined by referring recent papers including in vivo experiments for survival assay. The in vitro sample size was completed according to enable statistical analyses. The sample sizes of all animal experiments were described in each Figure legend
Data exclusions	No data was excluded from the analysis.
Replication	For all experiments, at least two biological replicates were analyzed in at least two independent experiments. All the replicates showed consistent reproducibility. Replication was as follow:<br>Fig 1a Data are representative of three independent experiments. Quantifications show the mean and SD of the three experiments.<br>Fig.1b Data is representative of three independent experiments.<br>Figure 1c. The effect of the drugs was observed in at least two independent experiments.<br>Fig 1d. Quantification of cell death upon the showed treatments was performed in two independent experiments.<br>Fig 2a, Fig2b and Fig 2c. Data is representative of two independent experiments in both NRAS mutated cell lines.<br>Fig 2d and Fig. 2e Data is representative of at least three independent experiments.<br>Fig 2f and 2g. Data is representative of two independent experiments in both NRAS mutated cell lines.<br>Fig 3a and 3b. Data represent the mean of five independent experiments.<br>Fig 3c These experiments were repeated twice. Each sample has a five biologically independent replicates in four different cell lines.<br>Fig 3d. Data was obtained from five independent experiments.<br>Fig 3e and 3f Means are calculated from three different experiments.<br>Fig 3g These experiments were repeated three times.<br>Fig 3h was replicated four times<br>Fig 4c-e Data was recollected from four independent experiments in two different cell lines.<br>Fig 5a and 5b Expression data was acquired from three independent experiments in each cell line.<br>Fig 5d Data is representative of two independent experiments.<br>Fig 6a and 6b. Data is representative of at least three independent experiments<br>Fig 6c. Data is representative of two independent experiments.<br>Fig 6d. The effect of the drugs was observed in at least three independent experiments.<br>Fig 6e. Data is representative of at least three independent experiments in three different cell lines<br>Fig 6f. Data represent the mean of five independent experiments.<br>Fig 6g. Staining was performed once.<br>Fig 6h and Fig 6i Data is representative of three independent experiments in two different cell lines<br>Fig 7a. Protein identification was performed from pooled samples from three independent experiments per condition and cell line.<br>Fig 7b-c. Data is representative of at least three independent experiments.<br>Fig 7d-Fig 7i. Experiments were repeated at least twice.<br>Fig 7K. Data is representative of two independent experiments.<br>Fig 8a-Fig 8d. Data is representative of at least two independent experiments.<br>Supplementary Fig1a. Data is representative of at least two independent experiments.<br>Supplementary Fig 1b-dData is representative at least two independent experiments.<br>Supplementary Fig 2a-g. Data is representative of at least three independent experiments.<br>Supplementary Fig 3b Data was obtained form quintuplicates<br>Supplementary Fig 4. Data was recollected from four independent experiments in two different cell lines..<br>Supplementary Fig 6b and Fig 6c Data show the result of two independent experiments.<br>Supplementary Fig 6d-Fig. 6f. Data was obtained form three independent experiments<br>Supplementary Fig 6h. Data is representative of three experiments.<br>Supplementary Fig 7d and Fig 7c Data is representative of at least three independent experiments.<br>Supplementary Fig 8c Data is representative of two independent experiments.<br>Supplementary Fig 9b Data is representative of two independent experiments.
Randomization	Upon tumor development 50mm3 animals were assigned randomly to experimental and control groups. Each experiment mice were age- and sex-matched.
Blinding	In general, the investigators were blind at the time of experiment execution and data acquisition. In vivo studies mice were identified by individual numbering after group allocations. Determination of tumor growth and weight were considered as objective measures, not subject to bias. For histopathological and microscopical examinations, samples were blinded to ensure unbiased imaging and analyses.

Reporting for specific materials, systems and methods

We require information from authors about some types of materials, experimental systems and methods used in many studies. Here, indicate whether each material, system or method listed is relevant to your study. If you are not sure if a list item applies to your research, read the appropriate section before selecting a response.

Materials & experimental systems

n/a	Involved in the study
☐	☒ Antibodies
☐	☒ Eukaryotic cell lines
☒	☐ Palaeontology and archaeology
☐	☒ Animals and other organisms
☒	☐ Human research participants
☒	☐ Clinical data
☒	☐ Dual use research of concern

Methods

n/a	Involved in the study
☒	☐ ChIP-seq
☐	☒ Flow cytometry
☒	☐ MRI-based neuroimaging

Antibodies

Antibodies used

Rabbit polyclonal anti-p-ERK1/2 (Thr202/Tyr204) Cell Signaling Technology Cat# 9101, RRID:AB_331646

Rabbit polyclonal anti-p-BRAF (Ser445) Cell Signaling Technology Cat# 2696, RRID:AB_390721

Rabbit polyclonal anti-p-CRAF (Ser259) Cell Signaling Technology Cat# 9421, RRID:AB_330759

Rabbit monoclonal anti-p-CRAF (Ser338) (56A6) Cell Signaling Technology Cat# 9427, RRID:AB_2067317

Rabbit polyclonal anti-p-CRAF (Ser289/296/301) Cell Signaling Technology Cat# 9431, RRID:AB_561402

Rabbit polyclonal anti-MEK1/2 Cell Signaling Technology Cat# 9122, RRID:AB_823567

Rabbit monoclonal anti-p-MEK1/2 (Ser221) (166F8) Cell Signaling Technology Cat# 2338, RRID:AB_490903

Rabbit polyclonal anti-AKT Cell Signaling Technology Cat# 4685, RRID:AB_2225340

Rabbit polyclonal anti-p-AKT (Ser 473) Cell Signaling Technology Cat# 4060,RRID:AB_2315049

Rabbit polyclonal anti-p-PKA substrate Cell Signaling Technology Cat# 9624, RRID:AB_331817

Rabbit monoclonal anti-cleaved Caspase 3 (Asp175) (5A1E) Cell Signaling Technology Cat# 9664, RRID:AB_2070042

Mouse monoclonal anti-Cyclin D1 (DCS6) Cell Signaling Technology Cat# 2926, RRID:AB_2070400

Rabbit monoclonal anti-PFKFB2 (D5I5F) Cell Signaling Technology Cat# 13029, RRID:AB_2798092

Rabbit monoclonal anti-p-PFKFB2 (Ser483) (D4R1W) Cell Signaling Technology Cat# 13064, RRID:AB_2798107

Mouse monoclonal anit-GST (26H1) Cell Signaling Technology Cat# 2624, RRID:AB_2189875

Rabbit polyclonal anti-caspase 6 Cell Signaling Technology Cat# 9762, RRID:AB_10829240

Rabbit polyclonal (D-21) anti-SOS1 Santa Cruz Biotechnology Cat# sc-259, RRID:AB_2270724

Goat polyclonal anti human SOS2, R and D Systems Cat# AF6260, RRID:AB_10643258

Rabbit polyclonal anti-14-3-3ß (K-19) Santa Cruz Biotechnology Cat# sc-629-G, RRID:AB_630820

Rabbit polyclonal anti-ERK2 (C-14) Santa Cruz Biotechnology Cat# sc-154-G, RRID:AB_631459

Mouse mooclonal anti-AIF (E-1) Santa Cruz Biotechnology Cat# sc-13116, RRID:AB_626654

Goat polyclonal anti-PARP (A-20) Santa Cruz Biotechnology Cat# sc-1562, RRID:AB_632158

Rabbit polyclonal anti-MKP-1/DUSP1 (V-15) Santa Cruz Biotechnology Cat# sc-1199, RRID:AB_2246131

Rabbit polyclonal anti-PFK-1 (H-55) Santa Cruz Biotechnology Cat# sc-67028, RRID:AB_2163019

Goat polyclonal anti-Lamin A/C (N-18) Santa Cruz Biotechnology Cat# sc-6215, RRID:AB_648152

Mouse monoclonal anti-NRAS (F155) Santa Cruz Biotechnology Cat# sc-31, RRID:AB_628041

Rabbit polyclonal anti-ARAF (C-20) Santa Cruz Biotechnology Cat# sc-408, RRID:AB_630882

Mouse monoclonal anti-BRAF (F-7) Santa Cruz Biotechnology Cat# sc-5284 HRP, RRID:AB_2721130

Mouse monoclonal anti-CRAF (E-10) Santa Cruz Biotechnology Cat# sc-7267, RRID:AB_628196

Mouse monoclonal anti-Melan A (M2-7C10) Thermo Fisher Scientific Cat# MA5-15237, RRID:AB_10980307

Rabbit polyclonal anti-PFKFB3 Proteintech Cat# 13763-1-AP, RRID:AB_2162854

Mouse monoclonal anti-GAPDH Proteintech Cat# 60004-1-Ig, RRID:AB_2107436

Rabbit monoclonal anti-Ki67 (SP6) Abcam Cat# ab16667, RRID:AB_302459

Mouse monoclonal anti-panRAS (RAS10) Millipore Cat# 05-516, RRID:AB_2121151

Rabbit polyclonal anti-p-PFKFB2 (Ser466) Millipore Cat# 07-1531, RRID:AB_11204022

Mouse monoclonal anti-ß-Actin Sigma-Aldrich Cat# A3854, RRID:AB_262011

ECL Anti-mouse IgG-HRP Sigma-AldrichCat# NXA931 RRID:AB_772209

ECL Anti-rabbit IgG-HRP Sigma-Aldrich Cat# NA934 RRID: AB_772206

ECL Anti-goat IgG-HRP Santa Cruz Biotechnology Cat# sc-2020 RRID: AB_631728

Goat anti-Rabbit IgG (H+L) Cross-Adsorbed Secondary Antibody, Alexa Fluor 594 Invitrogen Cat# A-11012 RRID: AB_141359

Goat anti-Mouse IgG (H+L) Cross-Adsorbed Secondary Antibody, Alexa Fluor 488 Invitrogen Cat# A-11001 RRID: AB_2534069

Validation

All the commercially available antibodies are validated by the manufacturers and validation data are available from the manufacturer websites and the references associted to each antibody:

Rabbit polyclonal anti-p-ERK1/2 (Thr202/Tyr204). Species Reactivity: human, mouse, rat, hamster, monkey, mink, D. melanogaster, zebrafish, bovine, pig, C. elegans. Application: Western Blot 1:1000, Immunoprecipitation 1:50, Immunofluorescence 1:200-1:800, Flow Cytometry 1:100-1:400. 7108 citations. https://www.cellsignal.com/products/primary-antibodies/phospho-p44-42-mapk-erk1-2-thr202-tyr204-antibody/9101

Rabbit polyclonal anti-p-BRAF (Ser445). Species Reactivity: human, mouse, rat, monkey. Application: Western Blot 1:1000. 72 citations. https://www.cellsignal.com/products/primary-antibodies/phospho-b-raf-ser445-antibody/2696

Rabbit polyclonal anti-p-CRAF (Ser259). Species Reactivity: human, mouse, rat, monkey, Xenopus. Application: Western Blot 1:1000, Immunoprecipitation 1:50. 123 citations. https://www.cellsignal.com/products/primary-antibodies/phospho-c-raf-ser259-antibody/9421

Rabbit monoclonal anti-p-CRAF (Ser338). Species Reactivity: human, mouse, rat, monkey. Application: Western Blot 1:1000. 219 citations. https://www.cellsignal.com/products/primary-antibodies/phospho-c-raf-ser338-56a6-rabbit-mab/9427

Rabbit polyclonal anti-p-CRAF (Ser289/296/301). Species Reactivity: human, mouse. Application Western Blot 1:1000.22 citations. https://www.cellsignal.com/products/primary-antibodies/phospho-c-raf-ser289-296-301-antibody/9431

Rabbit polyclonal anti-MEK1/2. Species Reactivity: human, mouse, rat, monkey, D. melanogaster. Application: Western Blot 1:1000, Immunoprecipitation 1:50. 549 citations. https://www.cellsignal.com/products/primary-antibodies/mek1-2-antibody/9122

Rabbit monoclonal anti-p-MEK1/2 (Ser221). Species Reactivity: human, mouse, rat, monkey. Application: Western Blot 1:2000 Immunohistochemistry (Paraffin) 1:50, Flow Cytometry 1:100. 151 citations. https://www.cellsignal.com/products/primary-antibodies/phospho-mek1-2-ser221-166f8-rabbit-mab/2338

Rabbit monoclonal anti-AKT. Species Reactivity: human, mouse, rat, monkey. Application: Western Blot 1:1000, Immunoprecipitation 1:100, Immunofluorescence 1:100, Immunocytochemistry (Paraffin) 1:200, Flow Cytometry (Fixed/Permeabilized) 1:100. 1535 citations. https://www.cellsignal.com/products/primary-antibodies/akt-pan-11e7-rabbit-mab/4685

Rabbit monoclonal anti-p-AKT (Ser473). Species Reactivity: human, mouse, rat, hamster, monkey, mink, D. melanogaster, zebrafish, bovine. Application: Western Blot 1:2000, Immunoprecipitation 1:50, Immunofluorescence 1:400-1:800, Immunohistochemistry

(Paraffin) 1:50 - 1:200, Flow Cytometry (Fixed/Permeabilized) 1:100-1:400. 8337 citations. https://www.cellsignal.com/products/primary-antibodies/phospho-akt-ser473-d9e-xp-rabbit-mab/4060. Additional validation was performed in our laboratory by Western Blot using AKT inhibitor MK-2206.

Rabbit monoclonal anti-p-PKA substrate (100G7E). Species Reactivity: all species. Application: Western Blot 1:1000, Immunoprecipitation 1:50, Peptide ELISA (DELFIA) 1:1000. 283 citations. https://www.cellsignal.com/products/primary-antibodies/phospho-pka-substrate-rrxs-t-100g7e-rabbit-mab/9624. Additional validation was performed in our laboratory by Western Blot using PKA inhibitor H-89.

Rabbit monoclonal anti-cleaved Caspase 3 (Asp175). Species Reactivity: human, mouse, rat, monkey. Application: Western Blot 1:1000, Immunoprecipitation 1:50, Immunohistochemistry (Paraffin) 1:2000, Immunofluorescence 1:400 - 1:1600, Flow Cytometry 1:6400. 4525 citations. https://www.cellsignal.com/products/primary-antibodies/cleaved-caspase-3-asp175-5a1e-rabbit-mab/9664

Mouse monoclonal anti-Cyclin D1 (DCS6). Species Reactivity: human, mouse, rat. Application: Western Blot 1:2000, Immunoprecipitation 1:100. 287 citations. https://www.cellsignal.com/product/productDetail.jsp?productId=2926

Rabbit monoclonal anti-PFKFB2 (D5I5F). Species Reactivity: human, monkey. Application: Western Blot 1:1000, Immunoprecipitation 1:50, Immunofluorescence 1:1600. 6 citations. https://www.cellsignal.com/products/primary-antibodies/pfkfb2-d5i5f-rabbit-mab/13029. Additional validation was performed in our laboratory by Western Blot with siRNA against PFKFB2 and PFKFB2 recombinant protein.

Rabbit monoclonal anti-p-PFKFB2 (Ser483)(D4R1W). Species Reactivity: human. Application: Western Blot 1:1000. 12 citations. https://www.cellsignal.com/products/primary-antibodies/phospho-pfkfb2-ser483-d4r1w-rabbit-mab/13064

Mouse monoclonal anit-GST (26H1). Species Reactivity: all Species (expected). Application: Western Blot 1:1000, Immunoprecipitation 1:200, Immunofluorescence 1:1600-16400. 143 citations. https://www.cellsignal.com/products/primary-antibodies/gst-tag-26h1-mouse-mab/2624. Additional validation was performed in our laboratory by Western Blot using a GST positive control.

Rabbit polyclonal anti-caspase 6. Species reactivity: human, mouse, rat. Application: Western Blot 1:1000. 97 citations. https://www.cellsignal.com/products/primary-antibodies/caspase-6-antibody/9762

Goat polyclonal (AF6260) anti-SOS2. Species Reactivity: human. Application: Western Blot. Additional validation was performed in our laboratory by Western Blot using siRNAs against SOS2.

Rabbit polyclonal anti-SOS1 (D-21). Species Reactivity: human, mouse, rat, pig, cow, dog, horse. Application: Western Blot 1:100-1:1000, Immunoprecipitation, Immunofluorescence 1:50-1:500, ELISA 1:30-1:3000. 17 citations. https://www.scbt.com/p/sos-1-2-antibody-d-21?requestFrom=search. Additional validation was performed in our laboratory using siRNAs against SOS1.

Rabbit polyclonal anti-14-3-3ß (K-19). Species Reactivity: human. Application: Western Blot 1:1000. 156 citations. https://www.scbt.com/p/pan-14-3-3-antibody-k-19?requestFrom=search

Rabbit polyclonal anti-ERK2 (C-14). Species Reactivity: mouse, rat, human. Application: Western Blot 1:100-1:1000, Immunoprecipitation (1-2 µg/100-500 µg total protein) , Flow Cytometry, Immunofluorescence (Immunocytochemistry) 1:50-1:500, ELISA 1:30-1:3000. 939 citations. https://www.scbt.com/p/erk-2-antibody-c-14?requestFrom=search

Mouse monoclonal AIF (E-1). Species Reactivity: mouse, rat, human. Application: Western Blot 1:100-1:1000, Immunoprecipitation (1-2 µg/100-500 µg total protein), Immunofluorescence 1:50-1:500, Flow Cytometry, ELISA (1:30-1:3000). 243 citations. https://www.scbt.com/p/aif-antibody-e-1?requestFrom=search

Goat polyclonal anti-PARP (A-20). Species Reactivity: mouse, rat, human. Application: Western Blot 1:100-1:1000, Immunoprecipitation (1-2 µg/100-500 µg total protein), Immunofluorescence (1:50-1:500), ELISA (1:30-1:3000). 26 citations. https://www.scbt.com/p/parp-1-antibody-a-20?requestFrom=search

Rabbit polyclonal anti-DUSP1/MKP-1. Species reactivity: human, mouse, rat. Application: Western Blot 1:100-1:1000, Immunoprecipitation(1-2 µg/100-500 µg total protein), Immunofluorescence 1:50-1:500, ELISA 1:30-1:3000. 56 citations. https://www.scbt.com/p/mkp-1-antibody-v-15?requestFrom=search

Rabbit polyclonal anti-PFK-1 (H-55). Species Reactivity: mouse, rat, human. Application: Western Blot 1:100-1:1000, Immunoprecipitation (1-2 µg/100-500 µg total protein) , Immunofluorescence 1:50-1:500, ELISA 1:30-1:3000. 10 citations. https://www.scbt.com/p/pfk-1-antibody-h-55?requestFrom=search

Goat polyclonal anti-Lamin A/C (N-18). Species Reactivity mouse, rat, human. Application: Western Blot 1:100-1:1000, Immunoprecipitation (1-2 µg/100-500 µg total protein) , Immunofluorescence 1:50-1:500. 124 citations. https://www.scbt.com/p/lamin-a-c-antibody-n-18?requestFrom=search

Mouse monoclonal anti-NRAS (F155). Species Reactivity mouse, rat, human. Application: Western Blot 1:100-1:1000, Immunoprecipitation (1-2 µg/100-500 µg total protein), Immunofluorescence 1:50-1:500. 221 citations. https://www.scbt.com/p/n-ras-antibody-f155?requestFrom=search. Additional validation was performed in our laboratory using siRNA against NRAS.

Rabbit polyclonal anti-ARAF (C-20). Species Reactivity: mouse, rat, human. Application: Western Blot 1:100-1:1000, Immunoprecipitation (1-2 µg/100-500 µg total protein), Immunofluorescence 1:50-1:500, ELISA 1:30-1:3000. 28 citations. https://

www.scbt.com/p/a-raf-antibody-c-20?requestFrom=search

Mouse monoclonal anti-BRAF (F-7). Species Reactivity: mouse, rat, human. Application: Western Blot 1:100-1:1000, Immunoprecipitation (1-2 µg/100-500 µg total protein) , Immunofluorescence 1:50-1:500, ELISA 1:30-1:3000. 272 citations. https://www.scbt.com/p/raf-b-antibody-f-7?requestFrom=search. Additional validation was performed in our laboratory using siRNA against BRAF and BRAF recombinant protein.

Mouse monoclonal anti-CRAF (E-10). Species Reactivity: mouse, rat, human. Application: Western Blot 1:100-1:1000, Immunoprecipitation (1-2 µg/100-500 µg total protein), Immunofluorescence 1:50-1:500, ELISA 1:30-1:3000. 106 citations. https://www.scbt.com/p/raf-1-antibody-e-10?requestFrom=search. Additional validation was performed in our laboratory by Western blot using siRNA against CRAF and CRAF recombinant protein.

Mouse monoclonal anti-Melan A (M2-7C10). Species Reactivity: human, mouse, non-human primate, rat. Application: Western Blot 1:50 - 1:100, Immunohistochemistry (Paraffin) 1:100 - 1:200, Immunofluorescence, Flow Cytometry, Immunoprecipitation 1:50. 24 citations. https://www.thermofisher.com/antibody/product/Melan-A-Antibody-clone-M2-7C10-Monoclonal/MA5-15237

Rabbit polyclonal anti-PFKFB3. Species Reactivity: mouse, rat, human. Application: Western Blot 1:1000-1:4000, Immunoprecipitation (0.5-4 µg/500 µg total protein), Immunohistochemistry/Immunofluorescence 1:50-1:500. 72 publications. https://www.ptglab.com/products/PFKFB3-Antibody-13763-1-AP.htm. Additional validation was performed in our laboratory using siRNAs against PFKFB3 and PFKFB3 recombinant protein.

Mouse monoclonal anti-GAPDH. Species Reactivity: human, mouse, rat, yeast, plant. Application: Western Blot 1:5000-1:50000, Flow cytometry, Immunofluorescence 1:20-1:200, Immunohistochemistry 1:50-1:500, Immunoprecipitation (0.5-4 µg/500 µg total protein). 1293 citations. https://www.thermofisher.com/antibody/product/60004-1-IG.html?ef_id=Cj0KCQjw--2aBhD5ARIsALiRlwDS_Xb4Z6cueRA9JuOmoQYneIXLC3n3SbzfJVeTeBY_Ys02KPTkN3saAqgGEALw_wcB:G:s&s_kwcid=AL!3652!3!459736943987!!!g!!!10950825775!106531320406&cid=bid_pca_aup_r01_co_cp1359_pjt0000_bid00000_0se_gaw_dy_pur_con&gclid=Cj0KCQjw--2aBhD5ARIsALiRlwDS_Xb4Z6cueRA9JuOmoQYneIXLC3n3SbzfJVeTeBY_Ys02KPTkN3saAqgGEALw_wcB

Rabbit monoclonal anti-Ki67 (SP6). Species Reactivity: human, mouse, rat. Application: Western Blot, Immunocytochemistry (Paraffin) 1:200, Immunofluorescence 1:250, Flow cytometry 1:1000. Knockout validated by the manufacturer. 1744 citations. https://www.abcam.com/Ki67-antibody-SP6-ab16667.html?gclsrc=aw.ds%7Caw.ds&gclid=Cj0KCQjw--2aBhD5ARIsALiRlwDwnxD4LpPjIctr8c6KdcOY44JkAIihxapgsAkiNmHQ3SXM5saKLUsaAmR3EALw_wcB

Mouse monoclonal anti-panRAS (RAS10). Species reactivity: human, rat, mouse. Application: Immunocytochemistry, Immunohistochemistry, Immunoprecipitation (1-4 µg/500 µg total protein), Western Blot 1:1000, ELISA, Flow cytometry. 53 citations. https://www.merckmillipore.com/ES/es/product/Anti-Ras-Antibody-clone-RAS10,MM_NF-05-516#anchor_REF

Rabbit polyclonal anti-p-PFKFB2 (Ser466). Species reactivity: human, mouse. Application: Western Blot (WB), ELISA.

Mouse monoclonal anti-ß-Actin. Species reactivity: all species. Application: Western Blot 1:20.000-1:50000. 1254 citations. https://www.sigmaaldrich.com/ES/es/search/a3854?focus=papers&page=1&perpage=30&sort=relevance&term=a3854&type=citation_search

Eukaryotic cell lines

Policy information about cell lines

Cell line source(s)	SKMel103 and SKMel147 cells were obtained from M. Soengas (CNIO, Madrid, Spain, ATCC). UACC903 cells were a gift from J. Trent (Tgen, Phoenix, AZ, USA). SKMel28, A375 and G361 were purchased from the American Type Culture Collection (ATCC, Manassas, VA, USA). NHEM were purchased from PromoCell (Heidelberg, Germany). Patient-derived cell lines, including MMLN1, MMLN9, MMLN10, MMLN14, MMLN16, MMLN23, MMLN24, MMLN30, MMLN31, MMGP3, MMSK8, MMSK22 and MMSK29 were derived from patients after tumor surgery. All samples were obtained upon the informed consent of the patients and the Vall d'Hebron Hospital ethical committee approval.
Authentication	SKMel103, SKMel147, SMEl28 and UACC903 cells were validated phenotypically, morphologically, genetically and biochemically. We assumed the authenticity of cells according to ATCC and PromoCell (G361/A375 and NHEM, respectively). Melanocytic origin of patient derived cells was validated through the expression of melanocytic markers, morphologically, phenotypically and genetically.
Mycoplasma contamination	All cell lines were tested negative for mycoplasma contamination.
Commonly misidentified lines (See ICLAC register)	No cell line listed by ICLAC was used in this study.

Animals and other organisms

Policy information about studies involving animals; ARRIVE guidelines recommended for reporting animal research

Laboratory animals	Athymic nu/nu, female 4-6weeks old were mantained under controled SPF housing normal (Dark/light cycles, temperature and humdity) conditions

Wild animals | The study did not involve wild animals

Field-collected samples | This Study did not involve sample collected from the field

Ethics oversight | Animal experiments were conducted and designed according to protocols approved by the Institutional Animal Care and Use Committee of Vall d´Hebron Institute of Research.

Note that full information on the approval of the study protocol must also be provided in the manuscript.

Flow Cytometry

Plots

Confirm that:

- [x] The axis labels state the marker and fluorochrome used (e.g. CD4-FITC).
- [x] The axis scales are clearly visible. Include numbers along axes only for bottom left plot of group (a 'group' is an analysis of identical markers).
- [x] All plots are contour plots with outliers or pseudocolor plots.
- [x] A numerical value for number of cells or percentage (with statistics) is provided.

Methodology

Sample preparation	Cultured cells treated with the indicated treatments for cell death analysis.
Instrument	FACSCalibur (Becton Dickinson)
Software	FlowJo v10
Cell population abundance	Relevant cell populations were not limited
Gating strategy	Annexin-V-FITC apoptosis kit (Biovision, Milpitas, CA, USA) was used following the manufacturer's instructions

- [x] Tick this box to confirm that a figure exemplifying the gating strategy is provided in the Supplementary Information.
