# Supplementary material for: BRAF activation by metabolic stress promotes glycolysis sensitizing NRASQ61-mutated melanomas to targeted therapy
Source: Nat Commun. 2022 Nov 19;13:7113. doi: 10.1038/s41467-022-34907-0 (PMC9675737; doi:10.1038/s41467-022-34907-0)
Supplement: Supplementary file 4 — Description of Additional Supplementary Files [file 41467_2022_34907_MOESM4_ESM.docx]

**Description of Additional Supplementary Files**

File Name: Supplementary Data 1

Normalized expression counts of the top 400 regulated genes in normal conditions (log2FC>0.265) between NRASQ61 (SKMel103 and SKMel147) and BRAFV600E (SKMel28 and UACC903) mutated cell lines.

File Name: Supplementary Data 2

Description: List of glucose metabolism-related genes showing the expression variations (log2FC) in BRAFV600E- and NRASQ61- mutant melanoma cells after glucose starvation (G.S.) for 1 h.

File Name: Supplementary Data 3

Description: Spectral counts and list of proteins identified in the His-tagged-PFKFB2 complexes by mass spectometry in NRASQ61 (SKMel103) and BRAFV600E (UACC903) mutant melanoma cells upon the indicated conditions. C=control, glucose starvation (G.S.) sorafenib (15µM) Sor.
