# Supplementary material for: BRAF activation by metabolic stress promotes glycolysis sensitizing NRASQ61-mutated melanomas to targeted therapy
Source: Nat Commun. 2022 Nov 19;13:7113. doi: 10.1038/s41467-022-34907-0 (PMC9675737; doi:10.1038/s41467-022-34907-0)
Supplement: Supplementary file 5 — Dataset 1 [file 41467_2022_34907_MOESM5_ESM.pdf]

	Normalized expression counts			
**SYMBOL**	**C SKMel103**	**C SKMel147**	**C SKMEL28**	**C UACC903**
A2M	2,3884866	2,0832445	7,079425	3,923067
AADAT	7,5125035	7,3413897	5,003174	2,57879
ABCA13	7,6071202	6,059283	2,841566	3,385447
ABCB5	1,9562294	2,7488742	6,977603	4,120774
ABCC2	1,2564511	1,7387755	6,719562	2,933769
ACP5	3,5310084	4,1475091	7,763299	5,542353
ADAM12	6,3969385	7,6310775	2,001329	4,04006
ADAMTS17	2,1052649	2,000693	5,527771	5,457884
ADAMTSL1	7,382897	5,8907651	1,802239	3,815721
ADGRL4	8,0660176	5,3882372	1,578743	2,778378
ADORA2B	6,4288498	6,4063607	2,92771	3,219811
AKAP12	9,5661851	8,5064822	8,565813	2,634878
ALCAM	9,3709628	8,5836724	6,998151	3,572517
ALDH1A1	2,056951	1,9598001	7,050457	3,28767
AMIGO2	6,6476712	6,7791567	2,837882	2,83475
ANKRD30B	0,8706825	1,1758777	6,548003	1,054695
ANXA1	11,42428	11,417347	10,61786	3,983074
ANXA3	8,1581601	7,9000298	5,499544	2,492076
AOX1	7,9787645	6,6999132	2,950226	4,407214
APOD	1,8954105	1,7244816	9,914458	4,437615
APOE	3,6080409	3,7650446	8,041622	6,074751
ARNTL2	8,1891746	7,1323914	2,809276	2,58962
ARPC1B	6,5897873	6,5388949	9,44284	8,860694
ASRGL1	2,0644033	1,9348553	5,466725	3,656378
ATP10B	2,3773449	1,8107929	6,35295	3,916364
ATP6V0D2	1,950184	2,4584937	7,02589	2,663029
AXL	9,8294174	9,0081476	4,032698	6,955031
BAIAP2L2	2,5291727	2,4440318	7,642919	4,581938
BAMBI	4,6015642	4,7093831	9,541801	6,400088
BASP1	7,5228443	7,9074055	2,619125	5,804797
BCAT1	8,3041472	8,1040577	2,248092	3,449307
BCL2A1	3,2535196	3,0972236	9,464515	2,637163
BEST1	2,1097644	2,0758204	8,331902	3,902855
BEX1	5,6940151	6,2863469	1,740664	2,00763
BIRC7	1,7858989	2,0565365	5,441421	4,617391
BMP7	2,0930721	2,0569538	5,533574	4,51041
BMT2	4,6685092	4,7628074	6,018387	9,073458
C10orf90	2,03547	1,5963856	8,332981	3,417853
C12orf75	8,4549358	7,862254	6,961891	2,714481
C1orf21	4,3938764	3,8914538	7,117834	8,983394
CA8	1,2605585	1,328007	6,167822	1,702031
CACNA2D4	7,7147302	7,7002726	3,976407	4,117252

CALB2	6,3001319	6,0591713	1,901709	2,881104
CAPG	3,8785739	2,9249099	10,55987	7,39031
CAPN2	10,022221	10,093393	9,463194	3,554071
CAPN3	1,6944129	1,7508942	8,809089	3,816009
CARD16	2,2422126	2,6442681	8,029991	4,635587
CASP1	1,1866795	1,5036828	6,549624	1,620718
CCL2	4,0184567	9,3145937	1,653633	4,335413
CCNA1	2,1882932	2,26775	5,477245	4,689383
CD200	2,5213441	2,5363032	7,880998	2,610788
CD24	7,516133	6,4895068	2,44327	4,03021
CDC123	9,6512778	9,4985212	8,701043	3,311408
CDH13	6,8833021	7,1659409	2,351355	3,648698
CDKN1A	8,2484069	8,5396426	3,303511	3,778977
CEACAM1	1,3250379	1,3827821	10,65155	2,680084
CEACAM5	1,9875041	1,849469	7,515831	2,511774
CELF2	6,8236919	3,201937	8,612379	9,649363
CHST11	6,2032747	7,5917135	10,71907	8,421072
CIAO2B	8,888758	8,8383904	7,639483	3,363806
CKMT1A	1,6302	1,4414663	5,409359	3,464921
CKMT1B	1,8815422	2,0808628	4,866947	4,292544
CLDN4	7,4420148	7,1515951	2,578268	3,707185
CLDND1	9,8041486	9,7009944	9,034213	3,18426
CLMP	7,1194884	6,6046	1,863308	3,467733
CLU	6,4044759	5,479665	1,975198	2,201811
COBL	2,1907766	2,4260008	5,342382	4,670063
COL12A1	6,705976	5,8646971	2,462531	3,066796
COL19A1	0,9433592	1,0881498	6,470512	1,460437
CPN1	2,2119488	1,86734	7,822556	3,3983
CPT1A	1,9171154	2,9056225	6,005167	4,088191
CRIM1	8,0462454	7,1373334	3,792424	4,175455
CSPG4	2,2715747	2,1935787	6,114374	4,035057
CST1	1,3183812	1,4668039	7,509895	1,698525
CTNNBIP1	3,4436625	3,558465	6,115125	7,126964
CTSH	1,2704608	1,5846597	8,169475	2,04408
CTSK	3,5626283	3,5000801	9,102358	5,423715
CYB5R2	9,1765784	8,9915656	4,874924	6,338397
CYP27A1	2,2251795	1,9084303	8,062262	2,225563
CYP7B1	1,7640794	1,8751753	6,368823	2,850587
DAAM2	3,0769994	1,9158118	7,888188	4,301029
DAPK1	3,8692643	4,8533754	8,195987	6,912133
DBNDD2	6,3773953	6,5616192	2,446956	2,124733
DCBLD2	11,327462	10,986204	10,07423	4,521016
DCT	3,1766712	4,0530686	10,99961	4,268297
DDAH1	8,745521	7,7117576	1,968612	5,482663
DIPK1B	2,9129827	3,2140533	7,44446	5,219109
DIS3	8,0359663	7,8671555	5,542145	3,187483

DKK1	9,031413	8,0101134	5,125229	3,356249
DKK3	7,5359165	8,1601326	1,872311	2,837659
DLGAP5	8,2648893	7,9707996	7,737018	1,833536
DMKN	8,3519089	4,5110308	3,369791	2,474909
DPYD	7,7872231	7,005289	4,818819	3,321327
E2F4	9,124006	8,8379923	7,245495	2,728857
EBNA1BP2	10,161703	10,346687	9,864892	3,804557
EIF1AY	2,0202412	1,8708059	5,358546	3,90669
ELL2	9,9198792	9,2830225	6,663474	4,564265
ELOVL2	1,9175472	3,6043665	6,538065	4,224387
ENOSF1	2,9891184	3,1938013	7,228294	4,816359
ENTPD1	1,6493236	1,2101918	7,74544	3,098344
ERBB3	1,4950797	1,4583497	7,706345	5,707092
ERRFI1	10,813961	10,239938	8,627687	2,856706
FABP6	6,4973844	5,6087787	1,958407	2,958819
FADS2	9,7084852	9,819468	6,109013	4,52728
FBLN5	2,0497541	1,9621196	4,963481	4,467768
FCRLA	2,772638	3,2771561	5,330975	6,355195
FERMT1	7,2069088	6,7281914	1,737459	2,744621
FLI1	5,9822709	6,0902637	2,15637	2,006695
FLRT3	7,6280672	8,2699394	4,430872	4,605358
FN1	3,8755452	6,0980852	10,61586	5,507381
FNDC10	1,7355726	1,8003144	5,174582	3,95282
FOSL1	9,8105219	9,2927166	7,589515	4,265382
FRAS1	2,0823886	2,0371801	3,929269	5,351724
FRMD4B	1,1809218	1,2445889	7,067331	2,005602
FXYD3	2,3810002	2,3800658	8,375057	4,695963
FYN	5,9920867	5,1591287	8,660367	8,757813
GABARAPL1	4,8586784	5,1238378	6,553989	9,450339
GALNT14	7,5189102	7,1156545	1,596172	2,722699
GAPDHS	2,7675746	2,5964133	9,466221	4,430237
GATA4	2,0788027	1,8355919	4,716015	4,623167
GDF15	2,957894	3,2906023	7,003268	5,66573
GDI2	9,7537146	9,6166513	9,35576	3,006027
GJA1	7,0165991	6,1689668	1,165093	4,665337
GJB1	1,4774185	1,6230654	8,614994	3,975574
GLB1L2	2,5357662	2,4765788	6,436807	4,318317
GLIPR1	10,502114	10,032409	5,35926	2,943844
GMPR	1,8344069	2,4496968	8,082026	3,718921
GNG7	2,016787	2,1023156	6,130713	3,802044
GOLPH3L	2,7608033	2,4625199	6,018226	6,335205
GPC4	1,452111	1,7201927	2,798824	5,966629
GPM6B	7,4405612	5,3946872	10,01465	8,346312
GPNMB	3,9681723	3,9072305	11,30689	6,69752
GPR143	1,7940016	1,6773395	8,715557	3,573093
GPRC5A	7,1121393	7,0264288	1,740628	3,3924

GPRC5B	4,1153769	3,7178549	7,28407	7,202237
GRAMD4	1,2084068	1,6680664	6,732558	2,010739
GSTM2	4,1115789	4,245208	7,75199	5,924344
GYG2	2,3905117	2,2567129	9,300227	3,436366
H1F0	6,100309	7,068342	1,941366	4,178599
H2AFJ	2,7737904	2,7575899	6,672362	4,901772
HACD2	9,5334668	9,2199846	8,588586	3,51069
HAS2	1,5676849	1,5398529	7,384734	2,991715
HENMT1	1,3209998	1,6081047	6,022044	2,610458
HERC5	1,2921713	1,4005922	5,488512	2,347188
HIGD1A	8,9970607	8,726965	8,459315	2,038508
HIST1H2BK	12,015001	11,904641	11,39453	5,822111
HIST1H3G	2,3927236	2,5265061	6,547487	4,013895
HIST1H3I	3,6853459	3,7038271	7,406859	5,518732
HIST1H4C	11,245745	10,736931	10,91131	3,895518
HLA-DMA	8,3615758	9,3144274	7,43733	2,779764
HLA-DPA1	8,6729636	9,1508175	7,931305	3,095998
HLA-DPB1	8,700135	9,3427468	5,481967	3,924897
HLA-DQA1	6,5784393	7,5229353	2,969529	4,220294
HLA-DRA	10,453114	11,156553	7,813494	2,205862
HLA-DRB1	8,8063	9,4076262	4,397914	3,832865
HTR2B	1,1695267	1,3999609	6,302243	2,537948
IDH2	4,2944947	4,4229456	7,019688	7,769183
IFI16	10,081389	10,453515	9,429063	3,525453
IFITM3	9,1568506	10,852593	6,482201	5,110587
IGFBP7	5,7230282	6,1656971	10,79775	7,700665
IGSF11	2,2208594	2,5881532	7,287388	4,391185
IL12RB2	2,0456406	2,0083534	6,080701	3,671803
IL16	1,6853424	1,5624708	6,412764	2,334146
IL1B	5,5752127	10,500035	5,189831	2,638486
IL24	9,1236403	9,0453799	2,961082	4,800015
IL31RA	6,2259714	5,9399617	1,565104	2,022799
IL7R	4,8923109	7,1573797	1,199773	1,901773
INHBA	5,6541076	8,1395047	2,149566	3,059949
IRF4	2,7184139	2,8089371	7,721889	5,64653
ITGA3	7,4738626	7,7117918	4,274931	2,044376
ITGA9	2,1815151	1,9743894	5,874613	4,386867
ITGB3	3,5087323	4,0362618	8,464812	4,59945
ITIH5	1,9771374	2,0114992	6,37322	4,494391
ITPRIPL1	5,7800493	5,7424891	1,792322	2,681687
JUN	7,506822	7,7618971	3,826357	4,661533
KAZN	2,410162	2,8688725	5,831306	5,027036
KCNAB2	2,8169538	3,2938535	10,33642	6,350214
KCNMA1	6,9990898	6,4345495	1,957895	3,630263
KIF20A	8,6827019	8,2073581	8,123326	2,092253
KNSTRN	9,6994694	9,5972706	8,452925	3,841187

KREMEN1	2,8708193	3,1485399	6,397148	6,134421
KRT18	8,1368659	7,4625058	5,628612	2,986974
KRT80	7,0379586	6,8143746	1,823305	2,765981
KRTAP2-1	9,518616	8,6473782	4,017652	6,721815
KRTAP2-2	9,5661325	8,5990624	4,337694	6,625742
KRTAP2-3	10,372551	9,2419376	4,351527	5,930303
LAMA1	1,7635336	1,7617249	7,993267	2,360658
LAMA4	3,611727	3,5370121	7,543902	5,71766
LAPTM5	7,3737382	7,9599395	3,958247	2,544466
LAYN	7,1467679	6,6911819	1,645165	2,788364
LCP2	2,6740918	3,0937411	6,631915	4,503759
LDHB	10,823175	10,73895	10,46103	4,285199
LGALS3	2,6690781	5,6719509	10,51681	5,42129
LHX8	3,880489	2,623137	7,551503	4,487263
LIMCH1	7,2898054	6,5891387	3,07278	3,523407
LINC00504	1,5179597	1,3355608	8,826666	2,847997
LPAR1	3,1292833	3,5957117	6,639138	5,233015
LRRC61	3,0773648	2,8550951	5,991756	5,492142
LRRK1	4,0996135	3,8566836	5,804748	7,534216
LURAP1	3,3855578	3,4658514	5,102027	7,394308
LXN	1,4808166	1,4187733	7,129687	1,857306
LY6K	9,5587951	9,8657723	2,74746	5,00766
MASP1	1,4169346	1,7385148	1,846716	7,266508
MBP	1,6113845	2,1436302	10,15234	6,090467
MCL1	9,6163922	9,6096367	8,349645	3,590061
MDK	9,4018506	9,2421651	4,042732	6,751997
MECOM	8,1150366	7,5259878	2,423748	3,742484
MERTK	1,2400917	1,9232559	6,430155	2,468812
MIR6809	2,5917248	2,4171334	6,717412	5,040105
MITF	5,3839211	4,8618709	10,52983	5,578739
MLANA	2,1363098	2,0228703	11,48168	5,067848
MLLT11	8,6282243	9,5305519	7,229906	3,473884
MMP17	3,0324534	2,8194081	7,395831	4,969713
MMP2	8,992264	9,5323425	4,000263	4,194947
MMP8	2,6574437	2,6471096	9,973102	2,78085
MRFAP1	9,0904978	9,1419625	7,952322	3,335873
MRPL42	7,9386515	7,7325306	6,574562	2,452009
MRPL51	9,6254351	9,5385647	8,977957	2,66886
MT1E	11,106459	11,145658	4,029009	6,09293
MT2A	12,2536	12,394902	12,00689	6,000379
MX2	5,9383935	6,788772	2,005402	3,203897
MYLIP	2,1503351	2,4986612	6,834133	4,70582
NCAM1	8,4389144	8,1913431	2,63077	4,092694
NDN	1,8388892	1,7010196	5,579579	4,092861
NEGR1	6,4204469	6,2139681	2,554596	2,949983
NEO1	2,6288852	1,3584467	6,483287	4,740939

NEXN	5,9653058	6,2037773	2,166464	2,216193
NFASC	6,865132	6,042476	1,895052	2,935411
NFATC2	2,9988254	3,5354133	8,3718	4,006348
NGDN	8,1896247	8,3329378	6,706659	2,969661
NGFR	9,388921	8,8333846	3,579751	5,41378
NID1	5,0027851	6,843565	1,344262	2,134921
NIFK	9,5043674	9,6196711	9,072544	3,274359
NR4A1	4,5400661	4,5422251	7,788261	6,593077
NR4A3	1,5909328	1,7674445	6,20832	2,270245
NRCAM	3,2775851	2,9287023	8,650586	5,149535
NREP	7,6061238	8,6750587	5,622898	3,860869
NRG1	6,9891813	7,2948282	2,999858	3,136045
NRP1	7,9686928	8,1488392	2,617455	4,371113
NTM	7,2719412	6,1517824	3,992223	2,497408
NUP188	7,8496997	7,4963757	7,02574	1,540163
OCA2	1,6950886	1,8001721	6,372312	2,969211
OCIAD2	5,8832798	5,949267	1,827161	2,838308
ODC1	10,324899	9,4223999	7,610093	2,679994
OSR1	2,6179974	2,28113	2,074013	8,777663
P3H2	1,4743721	1,9331469	7,310335	2,18011
PAK1IP1	8,7957653	8,8078292	7,603439	3,149258
PAQR3	9,1627795	9,1963059	6,846645	3,560245
PAX6	7,0545619	7,2485761	1,850341	2,397155
PCDH7	1,4797398	1,4080945	3,981204	5,583992
PCSK2	2,4464812	2,1328412	7,778687	3,89249
PDE1C	9,1486571	8,1139283	2,672702	3,830123
PDE4B	1,1985988	1,4692128	5,941173	3,736777
PDGFA	7,5216984	7,5707576	1,944808	5,289138
PDZD8	7,9562377	7,7299192	6,633612	1,796745
PHLDB2	5,8378425	6,0280838	2,446193	2,386841
PI15	1,944007	1,8921453	7,077672	3,53277
PLA1A	1,5560438	1,7366971	9,757475	3,690369
PLP1	4,1734556	3,9723835	10,08254	10,88631
PLXNC1	2,3994585	2,5287898	8,537567	3,742988
PMEL	1,5749542	1,9514782	11,33036	5,790334
PMEPA1	7,8586879	8,5470242	2,906758	4,3724
PMP2	1,2497901	1,2649267	7,285409	2,518091
PNKD	2,036233	2,2401986	5,20845	4,602101
PNP	9,0060121	8,7451525	2,135093	4,103267
PPP2R5A	4,0892362	3,9373097	5,951888	7,632541
PRDM7	1,883169	1,9041183	7,364312	3,946963
PRKACB	8,8880982	7,5455841	6,902182	2,567587
PRKG2	2,3216508	2,6235642	6,229174	4,153799
PRPF4	8,7895554	8,4964189	7,56894	2,551575
PRTFDC1	8,2406869	8,3214542	1,876936	4,155015
PSCA	2,9069329	2,9287598	7,619403	4,583903

PTPN3	1,2780559	2,1784264	5,761448	3,663243
PTX3	5,7739673	6,8807615	2,285506	3,377023
PYCARD	3,6169378	3,668896	8,364536	5,466941
QPCT	1,4767438	1,6886927	10,57563	3,831662
QPRT	2,3002491	2,1991168	6,58128	4,407224
RAB11FIP4	1,3182807	1,9238281	5,245915	3,132833
RABGGTB	8,7410161	8,8274264	7,687838	2,225024
RCN3	2,5298639	2,6381572	8,373882	4,812933
RFPL4A	6,0714284	4,8750664	1,696515	2,14632
RGMA	3,5512132	3,3523248	3,536706	8,662113
RNLS	2,0098515	3,053113	6,093455	4,094999
RPL3	11,598586	11,491914	11,34538	4,699666
RPS13	10,978781	11,20276	11,24119	4,234572
RPS24	10,780161	10,804327	10,36348	3,048959
RPS29	10,857602	10,555708	10,19736	3,797596
RPS4Y1	2,0832252	1,9593497	6,765337	2,657038
RUNX1	9,3773986	9,074389	3,744842	5,214606
RUNX3	2,8818678	1,6800735	7,590371	4,671629
RXRG	1,7688393	1,8176728	6,429646	3,192587
S100A1	2,7857573	2,5776142	8,837393	5,120762
S100A16	9,717067	9,7490368	3,334432	3,373626
S100A2	8,8227498	8,3083906	2,445147	4,776131
S100B	2,1202487	2,337748	8,974763	3,849389
S1PR1	9,4243656	6,4703096	2,558053	4,312109
SAMHD1	6,9576438	7,8845875	3,57901	2,341473
SCIN	1,1103934	1,3365894	7,813705	1,689856
SCML1	6,5250645	6,1901372	3,652206	2,42135
SCML4	1,7952942	2,1559607	6,949645	3,967944
SDC3	3,3158595	4,720365	10,19081	5,311617
SDC4	7,9895864	9,7820378	6,73796	3,706139
SDHD	8,4085609	8,0250288	7,84214	1,756813
SEMA6A	3,3584449	2,9185672	9,366811	6,711145
SERPINB2	8,4017233	6,6417034	3,820392	4,455344
SERPINB7	7,698509	6,9032099	1,471529	2,014193
SERPINE1	8,256551	7,3914543	2,340224	4,59567
SESN3	1,8160469	1,6012122	6,130704	2,8529
SGCD	4,1622115	3,3591236	8,449502	5,01128
SH3BGRL2	7,4436648	7,3803783	3,436942	4,317248
SIRPA	3,6120469	4,0793161	9,65273	4,644938
SKP2	9,1234632	8,8137049	7,455077	3,064237
SLC14A1	9,181407	8,9559452	1,943541	3,241776
SLC16A6	2,6003844	2,6821742	9,495928	4,635324
SLC17A9	3,2680981	2,9188338	7,443036	4,406044
SLC1A4	2,5690049	3,2441171	8,495247	4,025161
SLC22A23	3,6851728	3,1314746	4,603072	7,5311
SLC24A5	1,4606831	1,3706517	9,356255	3,949185

SLC27A3	1,8296317	1,6100937	7,35403	3,580628
SLC2A11	3,3928207	4,2925117	7,746928	5,679888
SLC38A1	8,1458301	8,1036717	5,844342	3,032401
SLC38A2	7,7249969	7,742814	6,182228	2,07891
SLC43A3	10,232658	10,197591	10,20429	3,088288
SLC45A2	1,6103505	1,541732	6,901659	3,119047
SLC7A8	1,3990682	1,4136415	9,522236	5,217554
SLCO2B1	1,6437774	1,4670385	5,249161	3,931478
SLFN11	7,1565606	6,5754813	2,611406	2,876302
SLIRP	10,164512	10,125609	9,463955	3,984183
SMAD5	9,0445361	8,9360226	8,6542	2,616009
SMN1	8,7481084	8,6638414	7,466809	2,282987
SMURF2	8,8329751	7,6395859	5,204931	3,162102
SNRPB2	8,8237246	8,7125026	8,122057	1,724571
SNX10	2,1874443	2,186329	7,899718	4,513533
SOX6	1,9350708	1,6564629	6,981182	4,005461
SPRR2D	2,9495017	1,5893906	6,505696	4,391286
SPSB1	4,7777857	5,8292953	9,305273	6,746189
SPTLC3	7,6806677	6,5724163	2,063568	2,839898
SRP72	10,060376	10,007932	9,563757	3,644241
ST6GALNAC2	2,8543751	3,4798604	8,527308	5,851315
ST8SIA6	1,1785343	1,1715205	4,205158	3,435754
STC1	8,6720316	8,0950446	4,921608	4,027015
STC2	6,7398326	7,0758206	2,33185	4,013335
STEAP3	7,9567408	8,0699975	3,072481	3,744754
SUB1	9,6746147	9,839867	8,996626	3,842294
SYNM	3,2838003	3,3660342	7,741384	5,410781
SYT1	6,6305053	5,2030899	1,5324	3,209877
TAF9	9,2668455	9,2458911	8,637942	2,891177
TBC1D16	4,9710855	4,5371743	8,349857	6,755659
TBL1X	6,9454685	6,6023157	4,430277	2,048005
TBX2	2,844115	4,0689252	7,447162	6,389397
TBX3	8,9476787	9,1725386	5,597083	3,671207
TFAP2A	5,1909006	5,3272092	7,77373	8,141016
TFB2M	8,482983	8,7376407	7,0588	2,754462
TGFB2	6,3790012	7,8012759	2,810354	3,21389
TGFBI	9,5709332	10,895371	2,233353	2,7909
TGFBR3	1,3510467	1,5941623	6,429444	2,691114
THBS1	10,273137	10,586952	3,851029	9,252245
TM4SF1	11,940011	11,538558	10,88456	3,608421
TMEM123	10,186233	9,9480971	9,536061	3,211626
TMEM14B	9,1566124	9,8679332	8,965041	2,729994
TMEM14C	8,375083	9,0795296	8,101143	2,296321
TMEM154	5,8838723	5,640405	1,660894	2,147003
TMEM167B	5,5247079	5,3619185	7,226383	9,219932
TMEM198	2,2187454	2,57944	3,597838	6,715799

TMPRSS15	9,292943	6,5044598	1,505258	1,86874
TMTC1	3,4036389	3,4241155	9,170309	6,125868
TNFRSF14	3,5952525	3,0790651	8,305027	5,27656
TNFRSF19	2,2401844	2,2440315	6,057889	3,627379
TOMM5	9,0521115	8,5343847	8,274102	2,514523
TPM1	7,6443118	9,3787037	4,746432	4,2655
TPM2	7,6027192	7,5592214	3,964626	3,799608
TRHDE	7,4915564	6,6883266	1,691832	2,770777
TRIB2	3,518497	3,6676489	8,304422	5,133617
TRIM63	2,0930742	1,9558487	6,91553	2,852031
TRPM1	2,1573919	2,5399275	6,558828	3,593766
TSPAN5	7,4362649	7,2703405	3,232982	4,244166
TSPAN7	4,3580428	1,5285813	6,941237	9,495432
TSTD1	2,1145316	2,1491133	6,820022	3,533224
TTYH2	3,471542	4,4228738	9,220986	6,891392
TXN	10,983113	10,770868	10,56288	4,467449
TXNL4B	7,6465333	7,7162171	5,69707	2,189073
TYR	2,0070741	1,7234423	8,716759	5,215867
TYRP1	1,9246362	1,5633843	7,077733	5,434416
UBE2T	9,3957072	9,7043753	8,651058	3,005816
UCP2	7,0745887	6,8533387	2,671267	3,379344
UQCRQ	9,5578777	9,614005	9,378646	2,547584
UTP4	8,9351608	8,7646218	7,475659	3,261098
VAMP8	1,3209112	1,0712548	5,177399	2,865504
VAV3	2,3827954	2,5517755	7,40282	3,218942
VEPH1	8,5452686	7,0359621	1,679753	3,00559
WDR66	7,1176557	6,5278362	2,609866	2,436748
XAGE1A	7,7676123	7,8398588	2,032782	3,748729
XAGE1B	6,7965269	6,7084215	1,400894	2,399234
ZEB1	8,1051833	7,5961796	3,548868	3,09511
ZNF146	9,194357	8,8155331	8,107813	2,84238
ZNF280B	1,6987439	1,6073157	6,434728	2,890828
ZNF518B	1,6751956	1,8235482	6,434838	3,815386
ZNF804A	6,2114701	5,0490481	1,847641	2,119463
ZNF883	6,595476	7,049677	3,170148	3,558465
ZNHIT6	6,6417508	6,6390043	4,945181	1,288366
