# Supplementary material for: BRAF activation by metabolic stress promotes glycolysis sensitizing NRASQ61-mutated melanomas to targeted therapy
Source: Nat Commun. 2022 Nov 19;13:7113. doi: 10.1038/s41467-022-34907-0 (PMC9675737; doi:10.1038/s41467-022-34907-0)
Supplement: Supplementary file 6 — Dataset 2 [file 41467_2022_34907_MOESM6_ESM.pdf]

GENE SYMBOL	Log2 FC G.S vs. C SKMel28	Log2 FC G.S vs. C UACC903
ALDOA	0,1813055	-0,1683008
ALDOB	0,30526605	-0,3223448
ALDOC	-0,2059192	0,15804464
DLAT	-0,2232457	-0,4629286
DLD	-0,0336277	0,71512988
ENO1	0,01492918	-1,1857557
ENO2	-0,0904763	-0,2513104
ENO3	-0,2028915	-0,0023544
FBP1	0,14815173	1,46538031
FBP2	-0,0897621	0,20011404
G6PC	-0,0954003	0,12260828
G6PC2	-0,0749561	0,05179656
G6PC3	-0,0562519	-0,8555394
G6PD	0,32660931	-0,0937212
GAPDH	-0,0957035	-0,6639756
GCK	-0,1065978	-0,6631748
GFPT1	0,10352196	-0,9218763
GFPT2	-0,2270588	-0,29893
GPI	0,25492854	-0,7019756
HK1	0,2944458	-0,4594668
HK2	0,12984763	-0,6093006
HK3	-0,2156331	-0,8952527
LDHA	-0,0173319	-0,1975397
LDHB	0,05721103	-0,7828902
LDHC	0,04181274	0,21182403
MPC1	-0,1941833	-1,0553286
MPC2	-0,1860513	0,03888135
PDHA1	0,08824411	0,51856182
PDHA2	0,25209886	-0,5018154
PDHB	-0,0310796	0,71375843
PDHX	-0,1272922	-0,5820978
PFKFB1	0,2850457	0,26660062
PFKFB2	0,35234691	0,05004925
PFKFB3	0,12914881	-0,3306627
PFKFB4	-0,2768203	0,35782065
PFKL	-0,1655813	0,5145444
PFKM	0,03872832	-0,2625451
PFKP	0,16667234	-0,0864978
PGAM1	0,03076702	-0,0033147
PGAM2	-0,066319	0,29838714
PGK1	-0,081604	0,4505472
PGK2	0,08854186	-0,1154432
PHGDH	0,08482617	-0,1430287
PKLR	-0,0658308	-0,9837244
PKM	0,11121802	-0,1391462

TIGAR	-0,1703881	-0,0765309
TPI1	-0,0556443	-0,1895579
