# Supplementary material for: BRAF activation by metabolic stress promotes glycolysis sensitizing NRASQ61-mutated melanomas to targeted therapy
Source: Nat Commun. 2022 Nov 19;13:7113. doi: 10.1038/s41467-022-34907-0 (PMC9675737; doi:10.1038/s41467-022-34907-0)
Supplement: Supplementary file 7 — Dataset 3 [file 41467_2022_34907_MOESM7_ESM.pdf]

5

	Spectral counts							
**Human Gene**	**C SKMel103**	**G.S. SKMEL103**	**Sor. SKMEL103**	**G.S. +Sor SKMEL103**	**C UACC903**	**G.S. UACC903**	**Sor. UACC903**	**G.S.+Sor. UACC903**
LMNA_HUMAN	126	100	115	86	268	256	242	203
PAIRB_HUMAN	112	92	110	121	79	97	92	106
SFPQ_HUMAN	90	95	88	103	102	105	89	89
BAG3_HUMAN	115	55	102	80	114	111	121	117
F262_HUMAN	47	135	43	93	53	58	51	47
SRC8_HUMAN	69	65	78	69	83	75	82	75
SF01_HUMAN	84	62	82	69	80	73	77	76
NONO_HUMAN	79	84	62	73	58	66	57	42
VIME_HUMAN	73	90	76	21	75	71	54	32
SF3A1_HUMAN	71	60	69	66	54	70	60	60
NUCB1_HUMAN	59	50	64	80	35	43	34	63
SNUT1_HUMAN	60	55	57	42	53	54	53	42
SF3B2_HUMAN	55	50	56	59	40	61	42	40
CCD50_HUMAN	53	29	53	47	47	46	55	53
AMRP_HUMAN	43	34	43	37	42	40	45	45
ATX2L_HUMAN	26	31	25	27	49	44	52	38
LC7L2_HUMAN	40	34	42	20	36	42	38	39
HSP7C_HUMAN	24	50	28	40	31	35	27	23
BIP_HUMAN	26	31	30	20	42	44	34	40
CKAP4_HUMAN	32	35	32	37	30	32	32	33
HNRPU_HUMAN	25	51	30	47	15	24	19	21
PDLI4_HUMAN	35	35	37	33	29	29	29	29
SERPH_HUMAN	17	47	19	20	26	40	22	30
DDX42_HUMAN	21	46	19	44	24	24	22	22
CE170_HUMAN	14	27	16	7	45	45	38	42
UBP2L_HUMAN	40	28	36	30	33	28	32	25
BCLF1_HUMAN	40	28	42	12	36	36	29	21
RS3_HUMAN	22	43	22	40	21	19	18	21
MCM3_HUMAN	15	44	13	36	23	22	22	18
ECM1_HUMAN	18	6	16	11	44	41	49	48
PSPC1_HUMAN	28	29	28	26	28	28	27	25
TBB5_HUMAN	23	48	20	38	17	18	14	15
RTRAF_HUMAN	26	19	31	24	29	32	31	30
NHRF2_HUMAN	26	17	28	26	29	27	29	29
GRN_HUMAN	26	18	21	19	31	30	33	29
ZC3H4_HUMAN	18	23	33	32	20	28	23	17
TR150_HUMAN	23	23	32	7	33	36	24	20
TBB4B_HUMAN	21	42	20	38	15	16	12	13
NSRP1_HUMAN	29	14	34	26	29	24	28	28
RBM25_HUMAN	25	29	25	32	19	21	21	14
CYR61_HUMAN	45	31	36	30	15	11	13	18
K2C1_HUMAN	33	15	23	40	16	7	27	39
RS3A_HUMAN	13	36	14	26	17	20	14	16
TOP1_HUMAN	17	41	14	33	7	18	8	9
EFTU_HUMAN	14	35	12	27	12	18	13	18
RS19_HUMAN	32	20	21	21	22	20	20	24
SF3A3_HUMAN	25	26	19	26	18	18	15	17
FIP1_HUMAN	21	13	21	8	30	33	29	18
HEXI1_HUMAN	16	12	12	16	32	22	27	32
MCRI1_HUMAN	34	17	33	28	15	20	18	15
WWTR1_HUMAN	27	16	32	19	20	17	20	21
PQBP1_HUMAN	21	19	19	22	21	20	18	21
EF1A1_HUMAN	17	24	19	25	21	16	17	13
CCDC6_HUMAN	24	22	30	34	7	9	12	22
SF3B1_HUMAN	7	53	1	39	2	2	3	5

TPX2_HUMAN	33	15	25	23	17	21	22	18
TMA16_HUMAN	17	14	17	17	26	21	23	23
SOX10_HUMAN	17	15	18	14	20	24	26	19
SF3B3_HUMAN	10	48	8	33	3	5	0	4
CPSF6_HUMAN	18	24	18	15	16	18	18	15
HNRPL_HUMAN	8	33	13	26	10	13	13	6
RS4X_HUMAN	6	36	9	31	6	10	8	11
CALM1_HUMAN	20	14	21	21	17	19	17	19
HS90B_HUMAN	14	17	19	18	13	17	21	19
YBOX1_HUMAN	28	14	21	16	18	14	24	17
ZO2_HUMAN	22	34	29	31	2	4	2	2
BRD4_HUMAN	19	16	19	21	17	17	18	14
TBA1B_HUMAN	0	30	13	23	11	15	0	16
HMMR_HUMAN	25	12	22	17	20	17	19	17
DHX15_HUMAN	6	50	5	20	2	2	2	3
WBP11_HUMAN	18	12	15	23	12	18	12	29
SNW1_HUMAN	15	15	12	25	11	16	15	22
TPM4_HUMAN	20	13	26	18	14	17	11	17
VGLL4_HUMAN	16	8	14	15	20	22	20	22
TJAP1_HUMAN	17	9	16	15	20	21	19	19
SF3B4_HUMAN	19	21	17	10	18	16	11	11
K1C10_HUMAN	20	8	15	21	14	12	18	28
RBM27_HUMAN	15	13	13	13	20	15	18	19
HS90A_HUMAN	13	19	22	21	6	11	12	12
SC16A_HUMAN	16	6	12	5	25	25	26	16
MAX_HUMAN	16	9	18	16	17	16	17	18
DNJA1_HUMAN	16	10	14	13	14	17	17	24
PALLD_HUMAN	14	11	11	10	23	20	15	17
MFAP1_HUMAN	17	13	18	23	12	12	13	13
RS5_HUMAN	15	17	18	14	11	16	13	10
PPIL4_HUMAN	12	22	11	12	10	16	11	12
TBA1C_HUMAN	11	32	13	24	0	13	0	0
CCDC9_HUMAN	18	8	19	14	16	14	14	20
RS17_HUMAN	15	21	9	14	11	17	10	10
NUFP2_HUMAN	7	6	6	7	19	21	26	20
FL2D_HUMAN	15	9	18	11	19	15	16	13
CPSF7_HUMAN	12	18	11	13	13	14	10	11
SMAP_HUMAN	19	14	19	16	9	10	8	16
ZO1_HUMAN	11	27	15	16	6	6	6	2
CPSF5_HUMAN	10	21	13	13	8	14	6	9
TPM3_HUMAN	16	8	17	14	12	14	15	16
RS10_HUMAN	16	17	13	13	7	11	9	16
RCN1_HUMAN	16	12	17	13	9	16	11	12
U2AF2_HUMAN	10	20	9	16	8	11	7	10
LEF1_HUMAN	9	4	9	9	17	22	20	16
SRRT_HUMAN	8	23	7	19	4	12	5	8
LUC7L_HUMAN	19	12	18	6	12	12	14	14
RSRC2_HUMAN	17	8	14	14	13	13	13	17
RLA2_HUMAN	14	11	16	11	11	11	14	15
CASC3_HUMAN	15	10	15	11	12	11	15	14
MYL6_HUMAN	15	11	13	11	13	13	12	13
SRPK1_HUMAN	12	22	13	17	4	6	6	7
TSR1_HUMAN	5	28	6	21	3	6	3	2
GPTC4_HUMAN	11	7	6	3	21	22	20	10
LYAR_HUMAN	15	21	15	17	5	10	2	4
PR40A_HUMAN	9	16	12	18	10	12	8	3
RS14_HUMAN	12	14	10	12	12	12	9	11
HXC4_HUMAN	16	7	18	9	14	12	12	14
K1C9_HUMAN	21	4	8	35	6	3	20	12

LUZP1_HUMAN	6	11	6	5	17	17	14	11
MCM5_HUMAN	0	30	0	22	2	4	1	3
PHF6_HUMAN	13	20	13	15	5	10	5	3
PP1RA_HUMAN	11	12	6	9	15	12	15	10
TYY1_HUMAN	14	11	15	8	12	11	10	12
RL23A_HUMAN	9	11	11	11	12	12	14	8
SMCE1_HUMAN	12	10	11	9	13	14	12	10
WDR33_HUMAN	6	5	12	10	19	16	14	8
SF3A2_HUMAN	16	13	13	9	10	12	9	8
SAFB1_HUMAN	14	9	14	13	13	12	10	6
SAFB2_HUMAN	14	8	14	14	12	12	9	7
CCAR2_HUMAN	8	29	7	0	5	6	4	4
SPS2L_HUMAN	6	15	7	6	8	10	11	12
TRA2B_HUMAN	17	7	15	9	14	13	10	8
UBAP2_HUMAN	5	12	7	9	12	13	10	8
K22E_HUMAN	9	4	5	24	6	3	9	27
SR140_HUMAN	4	20	6	20	5	5	5	1
NUCB2_HUMAN	3	3	4	4	5	16	15	32
MYCBP_HUMAN	9	9	11	11	9	10	12	10
ELOA1_HUMAN	6	11	7	6	11	11	13	11
DDX3X_HUMAN	0	32	0	16	0	1	0	0
RS21_HUMAN	9	10	11	10	8	10	8	11
CEP55_HUMAN	8	5	11	6	9	11	16	15
HNRH1_HUMAN	4	19	3	13	6	8	4	4
OGFR_HUMAN	15	7	14	7	13	8	10	9
PBIP1_HUMAN	9	10	10	8	8	9	9	10
SAP_HUMAN	12	4	14	9	10	7	10	15
CT027_HUMAN	10	16	8	11	4	7	5	6
RL31_HUMAN	10	9	8	9	10	8	7	12
RS15A_HUMAN	6	16	6	9	6	7	5	7
SRBS2_HUMAN	6	5	5	3	17	13	11	13
ACTB_HUMAN	0	21	4	11	3	4	3	5
TNIP1_HUMAN	14	5	14	7	10	10	10	9
CKS2_HUMAN	11	11	12	8	9	6	7	6
RS20_HUMAN	9	11	8	8	7	8	9	8
NPM_HUMAN	7	8	11	10	7	10	8	8
UBP7_HUMAN	0	27	0	10	1	2	1	2
SRRM1_HUMAN	6	2	8	25	5	7	6	14
DAP1_HUMAN	19	8	17	11	2	5	5	10
RS30_HUMAN	6	6	7	6	10	9	11	11
SF3B5_HUMAN	10	9	8	9	7	7	6	10
G3P_HUMAN	7	12	8	5	8	9	6	5
NOTC2_HUMAN	6	5	6	2	8	14	16	9
CDC37_HUMAN	10	7	10	10	6	7	9	8
ZN593_HUMAN	9	5	11	7	9	10	10	7
RS2_HUMAN	2	19	2	13	3	4	1	3
TBB2A_HUMAN	0	32	0	0	0	0	0	0
S30BP_HUMAN	9	9	10	7	7	7	8	6
KNOP1_HUMAN	8	6	6	9	12	10	8	6
RBM33_HUMAN	6	5	5	5	12	9	16	6
HS71A_HUMAN	0	13	0	13	9	11	0	4
CSTF2_HUMAN	11	6	13	9	7	5	10	6
PSMD9_HUMAN	10	6	9	4	9	9	7	12
DJB11_HUMAN	6	7	4	3	8	13	10	10
SCAF8_HUMAN	3	3	7	5	11	12	12	9
DBR1_HUMAN	6	15	5	12	4	3	3	4
DDX6_HUMAN	3	24	1	7	1	2	1	1
ATX2_HUMAN	1	13	3	9	6	6	6	5
IMDH2_HUMAN	0	18	0	22	1	0	0	2

HMGB1_HUMAN	13	4	13	9	6	5	11	8
NUCL_HUMAN	7	2	11	7	8	11	9	10
PRDX1_HUMAN	6	12	6	8	5	5	4	7
KINH_HUMAN	3	19	1	16	1	1	1	1
CSRP2_HUMAN	12	11	13	9	2	4	2	6
CBX3_HUMAN	9	7	8	7	8	7	7	6
NHP2_HUMAN	8	8	10	5	6	7	7	6
PRP16_HUMAN	0	21	0	12	0	2	0	1
HBA_HUMAN	6	9	7	7	5	6	5	8
CDK12_HUMAN	2	17	3	0	5	5	5	4
TYB4_HUMAN	8	5	10	10	5	5	8	7
CWC22_HUMAN	4	12	3	16	4	4	2	2
HEXI2_HUMAN	4	0	4	2	10	12	15	12
IF2B2_HUMAN	2	15	4	9	4	5	2	1
ERH_HUMAN	7	7	8	4	8	7	8	5
CCD97_HUMAN	6	6	11	5	6	7	7	6
NOB1_HUMAN	3	14	3	11	4	5	3	0
SASH1_HUMAN	1	0	0	0	12	15	14	13
ZN787_HUMAN	11	7	8	8	7	6	4	6
DYL1_HUMAN	5	6	5	5	6	7	9	9
PELO_HUMAN	4	15	6	10	4	1	1	1
AFF4_HUMAN	1	5	4	7	8	9	8	7
CD11B_HUMAN	0	16	3	16	0	0	2	0
SKP1_HUMAN	12	4	9	9	5	6	7	8
BCL7A_HUMAN	5	6	8	7	5	9	7	4
RPRD2_HUMAN	2	5	4	6	8	9	8	7
RUVB1_HUMAN	2	16	6	6	4	3	0	1
SPT2_HUMAN	11	3	9	5	7	6	10	8
SFRP1_HUMAN	8	8	7	7	3	6	5	7
PHF5A_HUMAN	7	7	6	6	6	6	7	6
MED15_HUMAN	6	8	7	5	6	7	4	6
RUVB2_HUMAN	5	17	3	12	1	1	0	0
NUSAP_HUMAN	11	8	9	10	4	2	6	2
SPF45_HUMAN	8	6	7	2	7	7	8	6
NDE1_HUMAN	7	1	7	5	9	9	10	7
RAVR1_HUMAN	3	16	2	7	2	4	0	2
DHX36_HUMAN	1	20	0	5	1	2	0	1
MED29_HUMAN	7	5	7	6	7	6	6	6
FRM4A_HUMAN	0	3	0	0	11	11	11	9
ALBU_HUMAN	8	0	4	14	15	2	5	7
LC7L3_HUMAN	6	6	6	6	5	7	5	6
HTSF1_HUMAN	5	8	5	8	5	3	5	5
WAC_HUMAN	5	8	5	6	5	6	5	4
RS27_HUMAN	4	9	3	7	5	4	5	5
PRC2C_HUMAN	3	1	0	5	6	7	8	19
PABP1_HUMAN	1	15	1	14	0	1	1	0
MATR3_HUMAN	0	17	1	3	3	3	1	2
MK01_HUMAN	0	17	0	11	0	0	0	2
RTCB_HUMAN	0	15	0	9	2	4	0	2
HOME3_HUMAN	11	8	6	5	6	5	4	4
S12A2_HUMAN	7	3	2	8	9	8	4	9
COXM2_HUMAN	5	4	7	6	6	7	6	6
TAGL2_HUMAN	5	7	8	5	5	5	4	5
SRPK2_HUMAN	0	10	0	8	4	3	4	7
CALU_HUMAN	4	0	6	2	8	9	10	10
HNRLL_HUMAN	2	13	3	9	0	2	2	3
DDX1_HUMAN	0	16	0	6	2	3	1	1
LARP1_HUMAN	0	9	3	8	4	4	6	2
FUBP1_HUMAN	9	3	13	7	4	5	5	4

KLF16_HUMAN	5	5	6	5	5	6	6	6
MELPH_HUMAN	5	3	5	4	7	7	6	9
PKHA5_HUMAN	4	4	5	3	7	9	6	6
RU2A_HUMAN	3	6	3	10	4	4	4	7
ILF3_HUMAN	2	16	3	9	0	0	0	0
PITH1_HUMAN	2	10	3	6	2	6	4	3
TAOK1_HUMAN	0	14	3	7	2	0	2	2
DJB12_HUMAN	8	8	7	8	1	5	2	4
TPM1_HUMAN	8	0	10	0	8	9	8	8
1433Z_HUMAN	5	8	6	7	4	3	3	4
MED19_HUMAN	9	3	7	5	6	8	6	4
ZGPAT_HUMAN	7	3	6	6	7	6	5	6
MED7_HUMAN	9	3	6	4	6	5	6	8
CNN2_HUMAN	7	2	7	2	11	5	9	3
ZFP91_HUMAN	7	6	6	6	5	5	4	3
MED9_HUMAN	6	3	7	3	6	7	7	5
RS12_HUMAN	6	4	8	5	5	5	5	5
RS11_HUMAN	1	10	2	6	5	4	1	3
SC31A_HUMAN	0	16	0	9	0	0	0	0
2B11_HUMAN	0	2	0	0	10	10	9	8
H2AV_HUMAN	6	4	4	5	5	7	5	6
TRI18_HUMAN	0	16	0	8	0	0	0	0
GPNMB_HUMAN	0	0	0	0	11	12	8	9
MAML1_HUMAN	7	3	7	5	4	5	4	8
TRA2A_HUMAN	7	2	8	3	8	9	3	4
CH60_HUMAN	4	5	4	12	2	4	1	6
U2AF1_HUMAN	4	6	4	4	6	4	4	5
CCNT1_HUMAN	3	6	2	5	5	4	5	6
RAI1_HUMAN	0	0	1	1	13	7	10	7
KAZRN_HUMAN	0	0	0	0	11	8	11	9
LTV1_HUMAN	9	5	8	7	3	3	5	2
MED8_HUMAN	7	4	6	4	5	6	6	3
RS18_HUMAN	5	9	4	6	1	3	3	3
COR1C_HUMAN	1	14	2	8	0	0	0	0
LMNB1_HUMAN	0	4	0	0	9	9	8	4
RAMAC_HUMAN	6	3	3	5	5	6	4	8
ZC3H8_HUMAN	5	4	6	6	3	4	5	5
RSSA_HUMAN	2	9	3	5	3	3	2	3
TIF1B_HUMAN	2	12	4	4	0	3	1	1
DDX47_HUMAN	1	15	1	6	0	0	0	0
ACPM_HUMAN	3	1	7	5	7	4	5	6
OTU7B_HUMAN	2	5	1	3	7	6	6	3
MTAP2_HUMAN	1	0	2	0	5	8	7	14
F263_HUMAN	0	13	0	10	0	0	0	0
TBB6_HUMAN	0	13	0	10	0	0	0	0
CALR_HUMAN	8	3	7	4	4	4	4	6
LASP1_HUMAN	6	1	7	3	7	3	7	6
IF5A1_HUMAN	4	7	5	4	3	2	1	6
RS13_HUMAN	4	6	4	4	4	3	4	4
SDF2L_HUMAN	4	3	2	2	7	7	5	6
1433T_HUMAN	3	7	5	4	2	2	2	6
ELAV1_HUMAN	3	12	1	7	0	1	1	1
PR38B_HUMAN	3	10	4	3	2	2	2	2
BRE1A_HUMAN	2	11	3	6	0	2	1	1
RL11_HUMAN	1	9	3	6	3	2	1	2
AKAP2_HUMAN	17	8	11	5	0	0	2	0
CRTC2_HUMAN	4	3	3	3	6	5	8	3
FA98A_HUMAN	2	8	2	7	2	3	2	2
LANC2_HUMAN	2	11	1	7	0	1	1	2

COF1_HUMAN	6	5	6	4	3	4	3	3
TF2AA_HUMAN	6	4	5	4	4	4	4	4
TFE2_HUMAN	6	4	4	6	4	5	4	2
B3A2_HUMAN	4	2	3	5	5	4	6	6
MARCS_HUMAN	4	2	7	4	6	3	4	5
RPC22_HUMAN	4	3	5	5	3	3	6	5
BUD13_HUMAN	6	3	8	4	3	4	4	3
RSRC1_HUMAN	5	2	4	4	5	4	4	7
SMD2_HUMAN	5	5	4	5	3	4	3	3
PI42C_HUMAN	2	10	1	4	1	1	2	3
PR38A_HUMAN	2	7	4	7	2	2	0	3
CAMP2_HUMAN	1	11	1	9	0	0	0	0
NEST_HUMAN	1	5	1	0	6	6	5	4
NRP1_HUMAN	1	10	1	9	0	1	0	1
SMRC1_HUMAN	1	10	0	9	0	3	0	0
BRE1B_HUMAN	0	12	0	7	0	1	0	0
STC2_HUMAN	9	7	9	7	0	0	1	0
PTMA_HUMAN	5	2	5	4	4	4	5	5
RFIP5_HUMAN	5	8	9	5	0	0	0	1
SQSTM_HUMAN	5	3	4	4	6	5	4	2
CKS1_HUMAN	4	4	5	4	3	4	4	3
COX5A_HUMAN	4	0	6	5	4	2	7	7
KLC2_HUMAN	3	7	3	5	0	2	3	4
RL9_HUMAN	1	10	1	4	2	1	0	3
DHX30_HUMAN	0	15	0	1	0	0	0	0
DDX18_HUMAN	0	13	0	5	0	0	0	0
ANXA2_HUMAN	0	12	1	6	0	0	0	0
AAPK1_HUMAN	0	11	2	6	0	0	0	1
STAU1_HUMAN	7	4	7	2	3	3	3	4
PP1RB_HUMAN	6	1	5	0	8	5	8	2
IN80B_HUMAN	4	1	4	1	5	6	7	5
RLA1_HUMAN	4	4	3	4	5	3	4	3
TFAP4_HUMAN	4	3	5	6	3	3	4	3
FUBP2_HUMAN	2	2	7	4	3	3	4	5
LEG1_HUMAN	2	3	3	3	5	4	5	4
HNRPF_HUMAN	1	8	2	6	2	4	0	0
STIM1_HUMAN	1	2	3	4	3	3	6	7
TACO1_HUMAN	1	6	1	5	3	4	1	4
HNRH2_HUMAN	0	8	0	7	3	4	0	0
TCF20_HUMAN	0	0	0	0	12	7	7	4
AHNK_HUMAN	0	0	0	0	5	7	12	6
LRRF2_HUMAN	7	2	6	4	6	2	4	3
CD44_HUMAN	4	3	6	5	2	3	3	4
RBM6_HUMAN	4	4	6	5	3	2	2	3
BCL7C_HUMAN	3	2	3	4	2	5	6	5
DNJA2_HUMAN	3	6	5	3	1	2	3	3
RM14_HUMAN	3	3	3	4	4	4	5	3
SOX13_HUMAN	3	0	3	0	7	8	6	5
MFGM_HUMAN	2	11	2	5	0	0	0	0
YLPM1_HUMAN	2	9	2	2	3	2	1	1
M4K4_HUMAN	0	8	0	7	1	2	2	1
SYNEM_HUMAN	0	0	0	0	11	8	6	4
SOX5_HUMAN	0	0	0	0	8	8	7	6
BICD1_HUMAN	7	3	8	2	3	2	3	4
CRTC1_HUMAN	7	3	5	3	1	3	5	5
NACAM_HUMAN	6	3	5	2	4	3	5	3
RL38_HUMAN	5	4	3	3	4	4	2	4
SSXT_HUMAN	5	3	3	3	4	5	3	4
STIP1_HUMAN	5	0	9	1	5	2	7	4

UT14A_HUMAN	4	5	4	3	3	4	3	1
NCOR2_HUMAN	3	0	3	0	7	6	9	3
WDR82_HUMAN	0	11	0	6	0	0	0	0
TANC1_HUMAN	0	5	3	4	1	3	2	5
TAOK3_HUMAN	0	5	0	4	3	4	3	4
RBMX2_HUMAN	4	3	4	2	4	4	4	3
C10_HUMAN	2	5	3	3	2	4	2	3
GOPC_HUMAN	2	3	2	4	3	4	3	5
CHERP_HUMAN	1	9	0	7	0	2	0	0
NSUN2_HUMAN	1	9	1	6	0	1	0	1
TCPB_HUMAN	0	12	0	3	0	0	0	0
LIPA1_HUMAN	0	2	0	3	5	5	7	3
DAXX_HUMAN	5	5	4	3	2	2	3	2
HINT1_HUMAN	5	3	4	3	4	3	3	3
CLASR_HUMAN	4	3	4	4	3	3	3	3
HSBP1_HUMAN	3	4	5	1	4	3	3	2
PPAC_HUMAN	2	3	5	4	1	2	2	6
RS16_HUMAN	0	7	2	5	0	2	1	2
SMRC2_HUMAN	0	7	3	6	0	3	0	0
DYL2_HUMAN	0	4	0	0	0	5	7	6
MPLKI_HUMAN	4	3	4	3	3	4	4	1
SMD3_HUMAN	3	4	3	4	2	3	2	3
SNIP1_HUMAN	2	1	5	3	4	7	2	2
CFA97_HUMAN	1	0	0	0	7	6	7	5
ZC3HD_HUMAN	1	3	2	3	4	3	5	2
AAKG1_HUMAN	0	9	0	5	0	0	0	2
HMGB2_HUMAN	8	2	10	7	0	0	3	0
CAAP1_HUMAN	4	1	4	0	7	4	5	2
CANB1_HUMAN	4	2	4	3	2	4	4	3
HDGF_HUMAN	4	0	9	3	1	3	4	4
PP12C_HUMAN	2	2	2	3	4	3	4	4
CPSF2_HUMAN	1	7	4	0	3	2	1	0
MECP2_HUMAN	1	2	1	1	4	5	5	4
PDIP2_HUMAN	0	10	0	2	0	1	0	1
NDUS6_HUMAN	6	4	4	4	1	1	2	3
ML12A_HUMAN	4	2	3	0	3	5	3	5
PUM1_HUMAN	4	1	5	3	4	4	3	2
RB6I2_HUMAN	4	2	7	6	0	0	0	6
CYTB_HUMAN	3	3	3	3	2	3	3	3
ELOB_HUMAN	3	3	3	3	5	4	1	1
RM12_HUMAN	3	3	5	5	1	1	3	2
RS28_HUMAN	3	2	3	3	3	4	3	3
CATC_HUMAN	2	6	4	3	0	2	0	2
SYNJ2_HUMAN	0	10	0	3	0	0	0	0
ZYX_HUMAN	7	1	7	2	2	2	4	3
T2AG_HUMAN	5	1	5	4	3	1	4	3
DPY30_HUMAN	4	2	2	2	6	4	2	2
PFD6_HUMAN	4	2	4	2	5	2	3	2
ANXA1_HUMAN	3	2	5	2	2	3	3	3
DCD_HUMAN	3	1	3	4	3	3	4	3
GPTC8_HUMAN	1	6	3	1	2	2	2	0
C1TM_HUMAN	0	10	0	2	0	0	0	0
CLPB_HUMAN	0	10	0	2	0	0	0	0
PP1G_HUMAN	0	9	0	4	0	0	0	0
DRB3_HUMAN	0	0	0	0	5	6	7	4
MEOX2_HUMAN	0	0	0	0	4	7	6	5
JUPI1_HUMAN	5	2	6	3	1	0	4	3
RANG_HUMAN	4	1	5	2	2	4	2	4
DKC1_HUMAN	2	4	3	4	2	2	1	1

NFIC_HUMAN	2	6	2	7	0	0	0	0
TRIP6_HUMAN	1	5	0	2	2	2	2	3
SELB_HUMAN	0	9	0	3	0	0	0	0
RIOK2_HUMAN	0	7	0	7	0	0	0	0
PDIA1_HUMAN	0	1	3	1	5	3	4	3
TBCA_HUMAN	5	0	4	1	3	4	4	4
NFYA_HUMAN	4	3	3	4	1	2	1	3
RT12_HUMAN	4	2	4	2	2	2	3	3
1433B_HUMAN	3	5	5	3	0	2	0	0
FKB1A_HUMAN	3	2	5	3	2	1	2	3
NOP16_HUMAN	3	3	2	3	4	3	2	0
PCBP1_HUMAN	3	7	0	4	1	1	0	0
REQU_HUMAN	3	0	3	2	2	4	6	3
CAPR1_HUMAN	2	2	3	6	0	1	0	6
CNBP_HUMAN	2	4	3	5	0	1	1	2
SF3B6_HUMAN	2	5	2	2	2	2	0	2
FXR1_HUMAN	1	7	1	4	0	0	0	1
RRP1B_HUMAN	1	6	0	3	2	1	1	1
ABCF2_HUMAN	0	9	0	2	0	0	0	0
2AAA_HUMAN	0	8	0	4	0	0	0	0
ILF2_HUMAN	0	8	1	3	0	0	0	0
LSG1_HUMAN	0	8	0	4	0	0	0	0
ENOA_HUMAN	0	6	5	3	0	0	0	0
ID4_HUMAN	0	0	0	0	5	3	6	6
CDV3_HUMAN	6	2	6	2	1	1	4	1
STMN1_HUMAN	6	1	7	2	2	1	3	2
ODFP2_HUMAN	4	1	3	1	3	3	3	4
S10AB_HUMAN	3	2	3	3	2	3	2	2
CWC25_HUMAN	2	2	2	1	2	3	5	2
PHRF1_HUMAN	2	1	4	0	4	4	4	1
RSMB_HUMAN	2	3	3	5	1	1	1	2
TOX4_HUMAN	2	4	2	3	2	2	2	0
ARMC6_HUMAN	1	6	1	3	0	1	0	2
PP1A_HUMAN	0	9	0	0	0	1	0	0
EDC3_HUMAN	0	7	0	2	1	0	0	2
2B1C_HUMAN	0	0	0	0	4	5	6	4
SSA27_HUMAN	6	4	7	3	0	0	0	0
CD2B2_HUMAN	4	2	4	3	1	2	1	3
CH10_HUMAN	4	1	4	1	4	3	2	2
YAF2_HUMAN	4	0	5	2	4	2	3	2
LRRF1_HUMAN	3	3	3	3	1	1	2	2
CRKL_HUMAN	2	2	2	2	1	3	2	4
IF4B_HUMAN	2	1	6	3	1	1	2	3
SETLP_HUMAN	2	2	4	2	2	2	2	2
THIO_HUMAN	2	4	3	2	0	2	1	2
ELOC_HUMAN	1	4	3	4	1	0	1	1
PPIA_HUMAN	1	4	1	1	2	3	1	2
RS29_HUMAN	1	3	2	2	2	2	2	2
HSP76_HUMAN	0	9	0	0	0	0	0	0
PP1B_HUMAN	0	9	0	0	0	0	0	0
ACOT9_HUMAN	0	7	0	4	0	0	0	0
EF2_HUMAN	0	7	1	3	0	0	0	0
EPS8_HUMAN	0	7	0	4	0	0	0	0
MARE2_HUMAN	0	7	0	0	0	2	1	1
NUDC1_HUMAN	0	7	0	4	0	0	0	0
SMCA4_HUMAN	0	6	0	4	0	0	2	0
UQCC3_HUMAN	0	2	4	1	4	2	0	3
K2C6B_HUMAN	23	0	0	7	0	0	0	10
PRR3_HUMAN	6	3	5	3	0	3	0	0

YBOX3_HUMAN	6	3	6	5	0	0	0	0
GLU2B_HUMAN	4	0	6	1	2	2	3	3
THOC7_HUMAN	4	2	4	3	1	2	1	2
KLC1_HUMAN	3	6	0	5	0	0	0	0
MAP4_HUMAN	3	1	3	0	2	3	4	3
MED30_HUMAN	3	2	3	2	2	2	2	2
RS27A_HUMAN	3	2	2	2	2	3	2	2
1433E_HUMAN	2	4	4	3	2	0	0	0
KIF5C_HUMAN	2	6	0	5	0	0	0	0
SRSF2_HUMAN	2	0	2	1	4	4	3	3
CAVN1_HUMAN	1	0	1	7	1	2	0	6
GRP75_HUMAN	1	5	1	6	0	0	0	0
LOXL2_HUMAN	1	8	1	0	0	0	0	0
SMAD4_HUMAN	0	7	0	3	0	0	0	0
RBM26_HUMAN	0	5	0	4	0	1	0	2
RL30_HUMAN	0	5	1	2	0	3	0	1
S10AA_HUMAN	0	5	0	1	0	2	1	3
USP9X_HUMAN	0	4	0	6	0	1	1	1
RCN3_HUMAN	0	0	0	0	5	5	3	4
MAR1_HUMAN	0	0	0	0	4	3	5	5
K1C16_HUMAN	34	0	0	8	0	0	0	8
MED21_HUMAN	3	2	3	2	1	2	2	2
MI4GD_HUMAN	3	4	4	4	0	0	0	0
RS25_HUMAN	3	2	2	2	2	2	2	2
RU1C_HUMAN	3	2	3	3	3	1	1	1
RUXF_HUMAN	3	3	2	2	1	2	1	2
S10A6_HUMAN	3	1	2	2	1	3	3	3
SCNM1_HUMAN	3	0	2	1	4	3	4	2
TYB10_HUMAN	3	1	5	2	1	1	1	4
CHID1_HUMAN	0	8	0	0	0	0	0	0
SC24B_HUMAN	0	8	0	0	0	0	0	0
CDK9_HUMAN	0	7	0	2	0	0	0	0
CBL_HUMAN	0	6	0	2	0	0	1	1
DDX17_HUMAN	0	6	0	4	0	0	0	0
PI51A_HUMAN	0	6	0	4	0	0	0	0
K1C14_HUMAN	24	0	0	7	0	0	0	8
MT2_HUMAN	7	3	7	2	0	0	0	0
DNS2A_HUMAN	4	2	2	2	2	2	1	2
SODC_HUMAN	4	0	2	0	4	2	3	4
PFD2_HUMAN	3	2	3	2	1	1	2	2
TAF10_HUMAN	3	1	5	1	2	2	2	1
CETN2_HUMAN	2	0	1	1	4	3	3	3
MRP_HUMAN	2	0	2	0	4	2	4	3
CATIN_HUMAN	1	6	1	0	0	1	1	0
LSM12_HUMAN	1	2	2	3	2	2	1	1
PI42A_HUMAN	1	5	0	3	0	1	0	1
KC1E_HUMAN	0	6	0	3	0	0	0	0
MTREX_HUMAN	0	6	0	3	0	0	0	0
RL12_HUMAN	0	5	1	3	0	0	0	1
MYL6B_HUMAN	0	0	0	0	0	4	4	7
DKK1_HUMAN	6	2	7	2	0	0	0	1
RIPL1_HUMAN	6	1	4	2	0	0	1	5
HS71L_HUMAN	4	0	6	0	0	0	4	4
JUPI2_HUMAN	4	0	5	1	1	2	3	2
ARGL1_HUMAN	3	1	2	3	1	1	1	4
HNRH3_HUMAN	3	5	0	2	2	0	0	0
ETV6_HUMAN	2	0	2	0	5	2	4	1
F10C1_HUMAN	2	3	2	0	1	2	1	2
MMTA2_HUMAN	2	1	1	0	3	2	4	2

SSBP_HUMAN	2	1	2	1	3	2	1	3
US6NL_HUMAN	2	1	3	0	2	2	2	3
CTCF_HUMAN	1	5	1	2	1	0	0	0
ZN609_HUMAN	1	2	1	0	3	4	2	0
DDX5_HUMAN	0	7	0	0	0	0	0	0
SYFA_HUMAN	0	6	0	2	0	0	0	0
WDR6_HUMAN	0	6	0	2	0	0	0	0
RHG17_HUMAN	0	5	1	3	0	0	0	0
PHS_HUMAN	0	2	0	2	2	2	2	2
PAGE5_HUMAN	0	0	0	0	4	3	4	3
K1C13_HUMAN	0	0	0	0	0	0	0	14
K2C4_HUMAN	0	0	0	0	0	0	0	14
K2C5_HUMAN	10	0	0	4	0	0	0	9
MED4_HUMAN	5	0	3	2	2	3	2	1
CIR1_HUMAN	4	2	3	1	0	1	4	0
HNRPC_HUMAN	4	2	2	2	2	1	1	1
GID8_HUMAN	3	2	3	0	3	1	1	1
TXLNA_HUMAN	3	0	4	1	1	2	2	3
EF1B_HUMAN	2	1	2	3	1	1	2	2
PPIG_HUMAN	2	3	3	1	1	1	1	0
RBM8A_HUMAN	2	2	2	1	1	2	2	1
TCAL8_HUMAN	2	0	3	0	3	2	3	2
ACBP_HUMAN	1	0	2	0	1	2	5	3
HXD4_HUMAN	1	1	1	1	2	2	3	2
LARP7_HUMAN	1	3	0	1	1	2	1	2
MBB1A_HUMAN	0	6	0	1	0	0	0	0
AAKB1_HUMAN	0	5	0	1	0	2	0	0
FLNA_HUMAN	0	5	0	3	0	0	0	0
CTBP1_HUMAN	0	4	0	4	1	0	0	0
SWAHC_HUMAN	0	4	0	4	0	0	0	1
UBP15_HUMAN	0	4	2	3	0	0	0	0
SPY2_HUMAN	0	1	0	2	2	3	2	2
NFX1_HUMAN	0	0	0	0	4	3	3	3
CTGF_HUMAN	6	0	4	0	4	0	3	1
PINX1_HUMAN	5	2	3	0	0	2	1	2
SPIN1_HUMAN	5	3	3	3	0	0	0	0
BCL9L_HUMAN	4	0	3	2	1	1	2	3
BASP1_HUMAN	3	0	7	5	0	0	0	0
BOD1_HUMAN	3	0	0	0	3	3	3	3
ENY2_HUMAN	3	0	3	2	2	2	1	2
PDZ11_HUMAN	3	3	2	1	0	1	1	1
NDKM_HUMAN	2	1	1	1	2	2	2	2
T22D1_HUMAN	2	1	1	3	0	0	2	4
TIPIN_HUMAN	2	0	2	2	2	2	2	2
CC85C_HUMAN	1	1	0	0	3	2	2	3
HOOK3_HUMAN	1	4	1	3	0	0	0	0
MED1_HUMAN	1	3	0	2	1	1	1	1
TF2H5_HUMAN	1	0	1	0	3	3	2	3
WDR5_HUMAN	1	2	1	0	3	2	1	1
ANM1_HUMAN	0	6	0	0	0	0	0	0
CTBP2_HUMAN	0	6	0	0	0	0	0	0
MYO1E_HUMAN	0	6	0	0	0	0	0	0
TRIM3_HUMAN	0	6	0	0	0	0	0	0
KC1D_HUMAN	0	5	0	2	0	0	0	0
M4K5_HUMAN	0	5	0	2	0	0	0	0
PESC_HUMAN	0	5	0	0	1	0	1	0
UBIP1_HUMAN	0	5	0	1	0	0	0	1
DDX24_HUMAN	0	4	1	2	0	1	0	0
ZCHC8_HUMAN	0	4	0	3	0	0	0	1

MCM2_HUMAN	0	3	0	6	0	0	0	0
RL23_HUMAN	0	2	2	2	1	1	1	1
PEG10_HUMAN	0	0	0	1	3	3	2	3
ZEB2_HUMAN	0	0	0	0	4	4	4	0
HXA3_HUMAN	0	0	0	0	3	2	3	4
HDAC4_HUMAN	0	0	0	0	2	3	5	2
ATIF1_HUMAN	5	0	0	1	3	3	2	2
FOSL1_HUMAN	5	1	4	1	1	1	1	1
HSPB8_HUMAN	4	3	4	1	0	0	0	0
TAF7_HUMAN	4	1	4	0	0	3	1	1
ZEB1_HUMAN	3	1	3	2	0	0	3	1
CHP1_HUMAN	2	2	1	2	0	2	0	2
IRF2_HUMAN	2	0	4	1	2	1	1	2
PHC2_HUMAN	2	0	0	0	5	2	3	1
CCNK_HUMAN	1	3	1	1	1	1	1	0
KHDR1_HUMAN	1	2	1	3	1	0	0	2
LGUL_HUMAN	1	1	3	1	1	1	2	1
TPIS_HUMAN	1	3	0	1	1	1	1	1
DJC10_HUMAN	0	5	0	1	0	0	0	0
KPYM_HUMAN	0	5	0	1	0	0	0	0
TBL1R_HUMAN	0	5	0	0	1	0	0	0
NOSIP_HUMAN	0	4	0	3	0	0	0	0
CHD7_HUMAN	0	0	0	0	5	1	3	2
AMOL1_HUMAN	0	0	0	0	3	6	1	1
RELL1_HUMAN	0	0	0	0	2	4	3	2
SBSN_HUMAN	0	0	0	0	2	2	5	2
HSP72_HUMAN	0	0	0	0	0	11	0	0
PMEPA_HUMAN	4	2	3	3	0	0	0	0
SURF2_HUMAN	3	1	2	3	0	1	1	1
HAP28_HUMAN	2	0	3	0	2	2	1	2
MED10_HUMAN	2	0	1	0	1	5	2	1
SLTM_HUMAN	2	1	1	0	2	3	1	1
SPAS2_HUMAN	2	1	2	2	1	1	1	1
HDAC1_HUMAN	1	3	1	0	0	2	1	0
PRIO_HUMAN	1	2	1	1	1	1	1	1
CPSF1_HUMAN	0	5	0	0	0	0	0	0
MYH10_HUMAN	0	5	0	0	0	0	0	0
PI42B_HUMAN	0	5	0	0	0	0	0	0
SC23B_HUMAN	0	5	0	0	0	0	0	0
TFCP2_HUMAN	0	5	0	0	0	0	0	0
CGT_HUMAN	0	4	0	2	0	0	0	0
EI2BB_HUMAN	0	4	0	2	0	0	0	0
F120A_HUMAN	0	4	0	1	1	0	0	0
LDHB_HUMAN	0	4	0	2	0	0	0	0
PROF1_HUMAN	0	4	0	2	0	0	0	0
QTRT2_HUMAN	0	4	0	2	0	0	0	0
SMD1_HUMAN	0	4	0	2	0	0	0	0
SMUF2_HUMAN	0	4	0	2	0	0	0	0
WDR48_HUMAN	0	4	0	2	0	0	0	0
ACL6A_HUMAN	0	3	0	1	1	1	1	0
NED4L_HUMAN	0	3	0	3	0	0	0	1
BOLA2_HUMAN	0	2	2	2	0	0	1	1
CHD8_HUMAN	0	2	0	2	1	1	1	1
RL37A_HUMAN	0	2	1	1	2	1	0	1
TXND5_HUMAN	0	2	1	0	1	2	1	1
RRP15_HUMAN	0	1	1	0	2	2	2	1
S39AA_HUMAN	0	0	1	1	2	2	2	2
NFAC2_HUMAN	0	0	0	0	4	2	3	1
GAS7_HUMAN	0	0	0	0	3	2	2	3

CT45A_HUMAN	0	0	0	0	2	2	4	2
G3BP2_HUMAN	0	0	0	0	2	4	1	3
TF2LX_HUMAN	0	0	0	0	2	4	2	2
MT1E_HUMAN	5	0	5	0	1	1	1	1
JUN_HUMAN	4	1	5	2	0	0	0	0
F10A1_HUMAN	3	0	4	2	1	0	1	1
HBEGF_HUMAN	3	2	2	3	0	0	0	0
RBM7_HUMAN	3	2	2	2	1	0	0	0
RD23B_HUMAN	3	0	0	2	0	3	0	4
SLIRP_HUMAN	3	2	1	0	2	2	0	0
TIM13_HUMAN	3	0	3	1	1	1	2	1
CO4A1_HUMAN	2	0	2	3	1	1	1	1
HNRPK_HUMAN	2	3	1	2	0	0	0	0
MPP10_HUMAN	2	1	2	0	1	4	0	0
PKHF2_HUMAN	2	1	0	1	2	1	1	2
BORG5_HUMAN	1	2	1	0	1	1	1	1
H2B1C_HUMAN	1	3	2	0	0	1	0	0
RNPS1_HUMAN	1	2	1	0	1	1	1	1
ROA1_HUMAN	1	1	1	2	1	1	1	1
SMCA2_HUMAN	1	0	1	2	1	2	2	1
SUMO2_HUMAN	1	1	1	2	1	1	1	1
TE2IP_HUMAN	1	2	2	1	2	0	0	0
TFAM_HUMAN	1	0	2	3	0	2	0	2
TWSG1_HUMAN	1	0	1	1	1	2	2	2
PAK4_HUMAN	0	4	0	1	0	0	0	0
PRP6_HUMAN	0	4	0	0	0	0	0	1
PTN11_HUMAN	0	4	0	1	0	0	0	0
TLE1_HUMAN	0	4	0	1	0	0	0	0
CTND1_HUMAN	0	3	0	3	0	0	0	0
P5CR1_HUMAN	0	3	0	3	0	0	0	0
PPIL1_HUMAN	0	3	0	3	0	0	0	0
SEC13_HUMAN	0	3	0	2	0	0	0	1
ZN638_HUMAN	0	3	0	0	1	1	1	0
3MG_HUMAN	0	2	0	0	2	1	2	0
LGMN_HUMAN	0	2	0	0	2	1	1	1
PRC2B_HUMAN	0	2	0	4	1	0	0	0
M3K7_HUMAN	0	1	1	1	2	1	1	1
SIN3A_HUMAN	0	1	0	0	1	1	3	2
SRGP2_HUMAN	0	1	0	2	1	1	1	2
NGRN_HUMAN	0	0	2	0	0	1	1	5
CLCA_HUMAN	0	0	0	0	3	2	2	2
CLSPN_HUMAN	0	0	0	0	3	3	3	0
CALD1_HUMAN	0	0	0	0	2	3	2	2
HXA4_HUMAN	0	0	0	0	2	2	3	2
TBA1A_HUMAN	0	0	0	0	0	0	9	0
PA1B3_HUMAN	5	1	4	1	0	0	0	1
AKA12_HUMAN	4	0	6	2	0	0	0	0
FA83G_HUMAN	3	0	1	1	2	0	2	2
SDE2_HUMAN	3	0	2	0	3	0	3	0
BYST_HUMAN	2	3	0	2	0	0	0	0
CSRP1_HUMAN	2	0	1	4	0	1	0	2
F117B_HUMAN	2	1	1	0	2	0	3	0
GRL1A_HUMAN	2	0	3	2	0	1	0	2
HXB4_HUMAN	2	0	2	0	2	2	2	0
IRS1_HUMAN	2	2	3	1	0	0	0	0
MANF_HUMAN	2	0	2	2	1	0	1	2
SKT_HUMAN	2	0	0	0	2	2	2	2
ZHX3_HUMAN	2	1	1	1	2	1	1	0
ANKZ1_HUMAN	1	2	0	4	0	0	0	0

COX17_HUMAN	1	1	1	1	1	1	1	1
EDF1_HUMAN	1	1	2	1	0	1	1	1
ENSA_HUMAN	1	1	1	1	1	1	1	1
F168A_HUMAN	1	1	1	1	1	1	1	1
MED31_HUMAN	1	1	1	1	1	1	1	1
MZT1_HUMAN	1	0	2	1	0	1	2	2
OTUD4_HUMAN	1	1	1	1	1	1	1	1
PTMS_HUMAN	1	0	2	1	1	1	2	1
DPP9_HUMAN	0	4	0	0	0	0	0	0
MARK3_HUMAN	0	4	0	0	0	0	0	0
PCBP2_HUMAN	0	4	0	0	0	0	0	0
PP2AA_HUMAN	0	4	0	0	0	0	0	0
RLA0_HUMAN	0	4	0	0	0	0	0	0
DDX49_HUMAN	0	3	0	2	0	0	0	0
HDAC2_HUMAN	0	3	0	2	0	0	0	0
TBPL1_HUMAN	0	3	0	1	0	1	0	0
H2A1B_HUMAN	0	2	0	0	0	4	0	0
HTF4_HUMAN	0	2	0	0	2	2	0	0
LARP4_HUMAN	0	2	0	2	0	1	0	1
DVL3_HUMAN	0	1	0	0	2	2	2	0
SOSSC_HUMAN	0	0	2	1	1	1	1	2
ATN1_HUMAN	0	0	0	2	2	3	1	0
SRSF8_HUMAN	0	0	0	0	5	0	3	0
PO3F2_HUMAN	0	0	0	0	3	2	1	2
CO8A1_HUMAN	0	0	0	0	2	2	2	2
SOSD1_HUMAN	0	0	0	0	2	2	2	2
DNJB6_HUMAN	0	0	0	0	1	4	2	1
MAZ_HUMAN	4	1	1	2	0	0	2	0
EEA1_HUMAN	3	0	5	2	0	0	0	0
EHBP1_HUMAN	3	0	2	1	1	1	1	1
RERE_HUMAN	2	0	0	0	0	3	3	1
RP9_HUMAN	2	0	1	0	0	2	4	0
RYBP_HUMAN	2	0	3	0	1	1	1	1
TNR16_HUMAN	2	0	4	1	1	0	0	1
UBAD1_HUMAN	2	1	1	0	1	2	1	0
4ET_HUMAN	1	0	1	0	1	2	2	1
AR6P4_HUMAN	1	0	2	3	0	0	1	1
MZT2A_HUMAN	1	0	2	2	1	1	0	1
PRP4B_HUMAN	1	3	1	0	0	0	0	0
R3HD1_HUMAN	1	1	0	1	1	1	1	1
SDCG8_HUMAN	1	0	1	0	2	0	1	3
SRSF3_HUMAN	1	1	1	1	1	1	0	1
ANR17_HUMAN	0	3	0	1	0	0	0	0
CDC20_HUMAN	0	3	0	1	0	0	0	0
DCA13_HUMAN	0	3	0	1	0	0	0	0
LDHA_HUMAN	0	3	1	0	0	0	0	0
MAGAA_HUMAN	0	3	0	1	0	0	0	0
MCES_HUMAN	0	3	0	1	0	0	0	0
PDLI7_HUMAN	0	3	0	1	0	0	0	0
RBBP7_HUMAN	0	3	0	1	0	0	0	0
RUFY1_HUMAN	0	3	0	1	0	0	0	0
STRAP_HUMAN	0	3	0	1	0	0	0	0
WDFY1_HUMAN	0	3	0	1	0	0	0	0
WIPI3_HUMAN	0	3	0	1	0	0	0	0
ZNFX1_HUMAN	0	3	0	1	0	0	0	0
GEN_HUMAN	0	2	0	0	1	1	1	0
CCNT2_HUMAN	0	1	0	1	1	1	1	1
EF2K_HUMAN	0	1	0	3	0	1	0	1
TAB2_HUMAN	0	1	0	0	1	1	2	1

EF1D_HUMAN	0	0	3	0	0	2	1	1
RPA34_HUMAN	0	0	2	2	0	1	0	2
ACTC_HUMAN	0	0	0	7	0	0	0	0
STIM2_HUMAN	0	0	0	1	2	2	2	0
LZTS1_HUMAN	0	0	0	0	5	0	2	0
DRA_HUMAN	0	0	0	0	2	3	1	1
ENPL_HUMAN	0	0	0	0	0	4	0	3
COA4_HUMAN	4	0	4	0	2	0	0	0
MK_HUMAN	3	1	2	1	1	0	0	0
MTMR6_HUMAN	3	0	0	0	2	0	2	2
ZN691_HUMAN	3	0	1	1	0	3	1	0
CHCH2_HUMAN	2	0	2	0	1	1	1	1
CSMT1_HUMAN	2	0	2	2	0	0	0	2
GPTC2_HUMAN	2	0	1	0	2	0	3	0
HMGA1_HUMAN	2	0	3	0	1	0	0	2
NDUS5_HUMAN	2	0	3	0	0	1	0	2
YAE1_HUMAN	2	0	2	0	1	0	2	1
CD11A_HUMAN	1	0	0	0	3	2	0	1
MASU1_HUMAN	1	1	0	2	1	1	0	0
MED22_HUMAN	1	1	2	0	0	1	1	0
MIDUO_HUMAN	1	0	0	1	1	2	1	1
MIF_HUMAN	1	2	1	1	0	0	0	0
NU153_HUMAN	1	0	1	0	1	1	2	1
PA2GD_HUMAN	1	1	1	0	1	0	1	1
PARK7_HUMAN	1	1	1	0	0	1	1	1
PYM1_HUMAN	1	0	1	1	1	1	1	1
SGTA_HUMAN	1	1	2	2	0	0	0	0
TCP4_HUMAN	1	0	1	1	1	1	1	1
TEBP_HUMAN	1	0	3	1	1	0	1	0
AAMP_HUMAN	0	3	0	0	0	0	0	0
AAPK2_HUMAN	0	3	0	0	0	0	0	0
AATF_HUMAN	0	3	0	0	0	0	0	0
ASCC3_HUMAN	0	3	0	0	0	0	0	0
EHD2_HUMAN	0	3	0	0	0	0	0	0
ERP44_HUMAN	0	3	0	0	0	0	0	0
F120B_HUMAN	0	3	0	0	0	0	0	0
FBP1L_HUMAN	0	3	0	0	0	0	0	0
IDH3B_HUMAN	0	3	0	0	0	0	0	0
IMA3_HUMAN	0	3	0	0	0	0	0	0
PI51C_HUMAN	0	3	0	0	0	0	0	0
PLEC_HUMAN	0	3	0	0	0	0	0	0
RBBP4_HUMAN	0	3	0	0	0	0	0	0
STA13_HUMAN	0	3	0	0	0	0	0	0
1433G_HUMAN	0	2	0	2	0	0	0	0
ALKB2_HUMAN	0	2	0	2	0	0	0	0
CARM1_HUMAN	0	2	0	2	0	0	0	0
CI114_HUMAN	0	2	0	2	0	0	0	0
CSPG2_HUMAN	0	2	1	1	0	0	0	0
FABD_HUMAN	0	2	0	2	0	0	0	0
MYPT1_HUMAN	0	2	1	0	0	1	0	0
SFSWA_HUMAN	0	2	1	1	0	0	0	0
TF3C4_HUMAN	0	2	0	2	0	0	0	0
CRIPT_HUMAN	0	1	0	1	0	0	1	2
POP1_HUMAN	0	1	0	0	1	1	1	1
RPAB3_HUMAN	0	1	0	1	1	1	0	1
SC61B_HUMAN	0	1	0	1	1	0	1	1
U520_HUMAN	0	1	0	4	0	0	0	0
ACBD5_HUMAN	0	0	1	1	1	0	2	1
P2R3A_HUMAN	0	0	1	2	0	2	1	0

PRRX1_HUMAN	0	0	0	0	2	2	2	0
ERBB3_HUMAN	0	0	0	0	1	2	2	1
MNT_HUMAN	0	0	0	0	1	2	2	1
PCLO_HUMAN	0	0	0	0	1	2	1	2
A4_HUMAN	3	1	2	1	0	0	0	0
CENPU_HUMAN	3	2	0	1	0	0	0	0
AES_HUMAN	2	1	1	1	0	1	0	0
CK098_HUMAN	2	1	0	0	1	1	1	0
HXA5_HUMAN	2	1	1	2	0	0	0	0
PFD4_HUMAN	2	0	3	0	0	0	1	1
QCR6_HUMAN	2	0	0	2	1	0	0	2
UBL4A_HUMAN	2	0	1	1	0	0	2	1
ACTA_HUMAN	1	0	0	0	0	0	0	5
ASPH_HUMAN	1	2	0	1	0	0	0	0
BTF3_HUMAN	1	0	3	1	1	0	0	0
CETN3_HUMAN	1	0	1	0	1	1	1	1
CH059_HUMAN	1	1	1	0	1	0	1	0
CHAP1_HUMAN	1	0	0	1	1	1	1	1
HNRPD_HUMAN	1	0	3	2	0	0	0	0
MED11_HUMAN	1	1	0	1	1	1	0	0
NDKA_HUMAN	1	2	0	1	0	0	0	0
RM32_HUMAN	1	0	1	0	1	1	1	1
RRP36_HUMAN	1	0	1	0	1	1	1	1
AMMR1_HUMAN	0	2	0	1	0	0	0	0
FYCO1_HUMAN	0	2	0	1	0	0	0	0
IMA1_HUMAN	0	2	0	1	0	0	0	0
METK2_HUMAN	0	2	0	1	0	0	0	0
RTF2_HUMAN	0	2	0	1	0	0	0	0
RXRB_HUMAN	0	2	0	1	0	0	0	0
SMRD1_HUMAN	0	2	0	1	0	0	0	0
SRGP1_HUMAN	0	2	0	1	0	0	0	0
TKT_HUMAN	0	2	0	1	0	0	0	0
TR112_HUMAN	0	2	0	1	0	0	0	0
TRIO_HUMAN	0	2	0	1	0	0	0	0
PPIB_HUMAN	0	1	0	0	1	2	0	0
SP1_HUMAN	0	1	0	1	0	1	1	0
U5S1_HUMAN	0	1	0	3	0	0	0	0
ZCCHL_HUMAN	0	1	0	1	0	1	0	1
GPT11_HUMAN	0	0	1	4	0	0	0	0
NUCKS_HUMAN	0	0	1	0	2	1	0	1
RLA0L_HUMAN	0	0	1	2	1	0	1	0
DIDO1_HUMAN	0	0	0	1	2	1	0	1
HMGN2_HUMAN	0	0	0	1	0	2	1	1
ANR54_HUMAN	0	0	0	0	2	0	2	1
NIPBL_HUMAN	0	0	0	0	2	2	1	0
TCOF_HUMAN	0	0	0	0	2	1	1	1
CC85B_HUMAN	0	0	0	0	1	0	2	2
JHD2C_HUMAN	0	0	0	0	1	2	2	0
PIMRE_HUMAN	0	0	0	0	1	1	2	1
RING1_HUMAN	0	0	0	0	1	2	2	0
CAB45_HUMAN	0	0	0	0	0	3	1	1
HORN_HUMAN	0	0	0	0	0	0	4	1
